# RNA Pol II inhibition activates cell death independently from the loss of transcription

**DOI:** 10.1016/j.cell.2025.07.034

**Published:** 2025-08-15

**Authors:** Nicholas W. Harper, Gavin A. Birdsall, Megan E. Honeywell, Kelly M. Ward, Athma A. Pai, Michael J. Lee

**Affiliations:** 1 Department of Systems Biology, UMass Chan Medical School, Worcester, MA 01605, USA; 2 Program in Molecular Medicine and Department of Molecular, Cell and Cancer Biology, UMass Chan Medical School, Worcester, MA 01605, USA; 3 RNA Therapeutics Institute, UMass Chan Medical School, Worcester, MA 01605, USA; 4 Lead contact

## Abstract

RNA Pol II-mediated transcription is essential for eukaryotic life. Although loss of transcription is thought to be universally lethal, the associated mechanisms promoting cell death are not yet known. Here, we show that death following the loss of RNA Pol II activity does not result from dysregulated gene expression. Instead, it occurs in response to loss of the hypophosphorylated form of Rbp1 (also called RNA Pol IIA). Loss of RNA Pol IIA exclusively activates apoptosis, and expression of a transcriptionally inactive version of Rpb1 rescues cell viability. Using functional genomics, we identify the mechanisms driving lethality following the loss of RNA Pol IIA, which we call the Pol II degradation-dependent apoptotic response (PDAR). Using the genetic dependencies of PDAR, we identify clinically used drugs that owe their lethality to a PDAR-dependent mechanism. Our findings unveil an apoptotic signaling response that contributes to the efficacy of a wide array of anti-cancer therapies.

## In brief

When RNA polymerase II (RNA Pol II) is inhibited, the inhibition is sensed and signaled to mitochondria, leading to programmed cell death independently of the loss of RNA Pol II transcription activity.

## Graphical Abstract

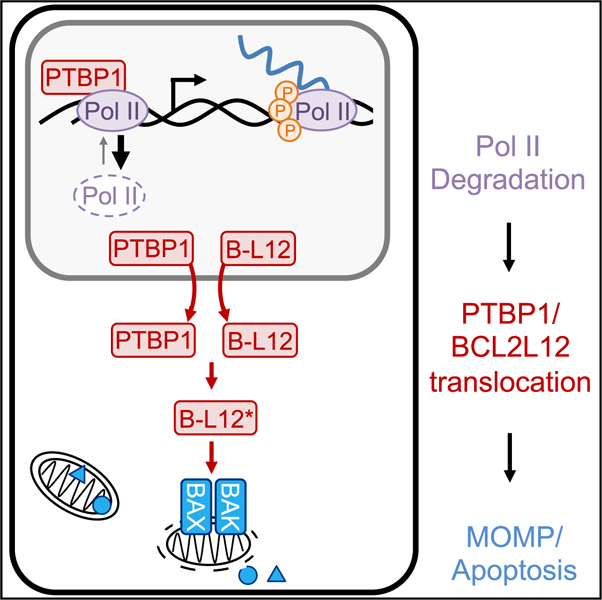

## INTRODUCTION

Accurate control of gene expression is critical for all cellular processes. For nuclear protein-coding genes, expression depends on transcriptional activity of RNA polymerase II (hereafter, RNA Pol II). RNA Pol II activity is considered a life-essential function, and prolonged inhibition of RNA Pol II is expected to be universally lethal.^1^ In recent years, drugs targeting the transcriptional machinery have been investigated as cancer therapies.^2^ However, despite the importance of RNA Pol II and clinical interest in drugs targeting transcription, the mechanisms of lethality following transcriptional inhibition remain unclear.

Lethality following RNA Pol II inhibition remains mechanistically uncharacterized largely due to the presumption that death in this context is not regulated. Regulated cell death refers to death induced by active signaling through specific effector enzymes or defined pathways.^3^ By contrast, transcriptional inhibition is generally assumed to cause death using passive mechanisms, and this begins with mRNA decay and the subsequent loss of protein, leading to some unavoidable catastrophic event. Cell death of this type is sometimes referred to as “accidental cell death,” highlighting the use of passive mechanisms, such as decay.^4^

In contrast with the notion that transcriptional inhibition causes accidental death, recent studies highlight that cells are remarkably capable of buffering against perturbations to the mRNA pool.^5–9^ For instance, in normal conditions, mRNA concentration scales with cell size, and variations in cell size are buffered by tuning RNA Pol II activity or mRNA decay to maintain stable mRNA concentrations.^7,10^ Likewise, following perturbations that reduce mRNA production, the effects are blunted by reduced mRNA degradation rates.^7,8^ Thus, if cells have the capacity to buffer against downstream effects of transcriptional inhibition, why do they instead die? To address this question, we aimed to formally characterize the mechanism(s) of lethality following RNA Pol II inhibition in different contexts.

Here, we make the discovery that cell death following RNA Pol II inhibition is not caused by general decay of mRNA and protein. Instead, death is activated by loss of RNA Pol II itself, specifically loss of the hypophosphorylated and non-transcribing forms of RNA Pol II, collectively referred to as RNA Pol IIA. We determine that lethality following loss of RNA Pol IIA is initiated by an apoptotic signaling response, and using chemogenetic profiling, we identify the mechanism by which levels of RNA Pol IIA are sensed and transmitted from the nucleus to the mitochondria to initiate apoptosis. Finally, we profile a panel of compounds, including clinically useful drugs, identifying several unrelated compounds that kill cells using an RNA Pol II degradation-dependent mechanism. Collectively, our results reveal the existence of an apoptotic pathway that senses RNA Pol IIA levels, not RNA Pol II transcription, triggering death when RNA Pol IIA levels become too low.

## RESULTS

### Transcriptional inhibition exclusively activates apoptosis

**Figure 1.**
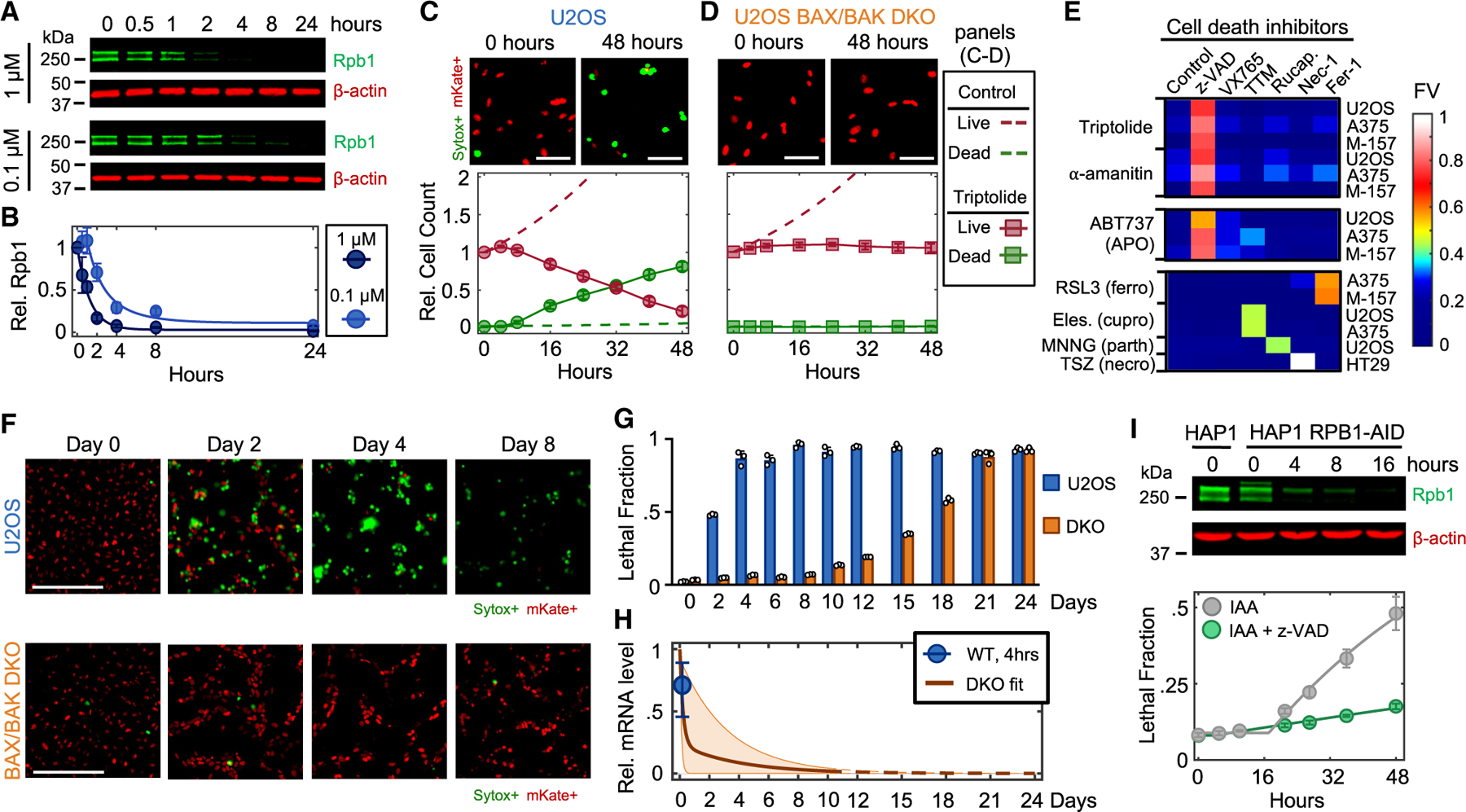
RNA Pol II inhibition activates apoptosis prior to passive decay of apoptotic mRNA and protein (A) Immunoblot of Rpb1 in U2OS following 1 or 0.1 μM triptolide (TRP). Blots representative of 3 biological replicates. (B) Quantification of (A). (C and D) Live/dead cell kinetics following 1 μM TRP in Nuc::mKate2-expressing U2OS cells. mKate+ = live; Sytox+ = dead. (C) U2OS. (D) U2OS^BAX−/−/BAK1−/−^ (BAX/BAK DKO). Scale bar, 100 μm. (E) Fractional viability (FV) following drugs ± pathway-specific inhibitors: z-VAD-FMK (z-VAD) inhibits apoptotic caspases; VX765 inhibits pyroptotic initiator, caspase-1; TTM inhibits cuproptosis (cupro); Rucaparib (Rucap.) inhibits parthanatos (parth); Nec-1 inhibits necroptosis (necro); and Fer-1 inhibits ferroptosis (ferro). RSL3, elesclomol (Eles.), MNNG, and TSZ are canonical activators of listed death pathways. M-157, MDA-MB-157. Heatmap scaled to the mean of 3 biological replicates. (F and G) Long-term response to 1 μM TRP. (F) Representative images from 3 biological replicates. Scale bar, 275 μm. (G) Lethal fraction (LF). (H) mRNA levels following 1 μM TRP, relative to untreated cells. mRNA was quantified for times in which lethality had not yet occurred (4 h for WT; 10 days for DKO). Range for DKO represents 50 longest/shortest half-life mRNAs. Dashed lines denote extrapolation from fit. For WT, the median, 25^th^, and 75^th^ percentiles are shown. (I) (Top) Immunoblot of Rpb1 in HAP1, or HAP1-RPB1-AID, following 500 μg/mL auxin (IAA, 3-indoleacetic acid). (Bottom) LF for HAP1-RPB1-AID cells +IAA, ±z-VAD. Data are mean ± SD of 9 biological replicates. For panels with error bars, data are mean ± SD of 3 biological replicates unless otherwise noted. See also Figures S1 and S2 and Tables S1 and S2.

To investigate transcriptional inhibition-induced cell death, we focused on triptolide and ⍺-amanitin, two well-validated transcriptional inhibitors that degrade RNA Pol II.^11,12^ We first measured levels of Rpb1, the largest essential subunit of RNA Pol II. As expected, U2OS cells exposed to triptolide rapidly and completely degraded Rpb1, and Rpb1 degradation kinetics could be titrated in a drug-dose-dependent manner (Figures 1A, 1B, and S1A). To validate that Rpb1 protein levels could be used to interpret the transcriptional state in the context of triptolide, we also measured incorporation of 5-ethynyl uridine (EU) into nascent RNAs, which confirmed the kinetics of transcriptional inhibition following triptolide exposure (Figures S1B and S1C). Similar dose and time behavior was observed following exposure to ⍺-amanitin (Figure S1D).

To characterize death in response to RNA Pol II inhibition, we used 1 μM triptolide, which completely degraded Rbp1 within ∼4 h (Figures 1A and 1B). Using the STACK assay to quantify live and dead cells,^13^ we observed that live cells proliferated normally for only 4 h following triptolide exposure, followed by a complete loss of proliferation, coinciding with the initiation of cell death (Figure 1C). Death kinetics at lower doses of triptolide were similar, but with a delay in death onset that matched the later degradation of RNA Pol II (Figure S1A). Similar results were observed with ⍺-amanitin, suggesting that rapid onset of lethality is a general feature of drugs that degrade RNA Pol II (Figure S1D) and that cell death is the primary phenotypic outcome of complete transcriptional inhibition.

Since loss of RNA Pol II initiated cell death more rapidly than expected following transcriptional inhibition, we next aimed to characterize which death mechanism(s) are activated. At least 14 forms of regulated cell death have been characterized.^14–16^ We reasoned that if cell death following loss of transcription resulted from passive protein loss, rather than active signaling, then inhibition of any single death pathway should fail to rescue viability. To test this, we genetically or chemically inhibited each death pathway, one at a time, and observed the effect on cell death following triptolide exposure. To inhibit apoptosis, we knocked out the mitochondrial pore-forming proteins, BAX and BAK (*BAK1*).^17,18^ Remarkably, BAX/BAK double-knockout (DKO) cells were completely resistant to the lethal effects of triptolide (Figures 1D and S1E). While BAX/BAK DKO cells did not die, these cells were rendered completely non-proliferative within 4 h of triptolide exposure, suggesting that triptolide had completely inhibited transcription (Figures 1D, S1F, and S1G). Triptolide-induced death was also suppressed using the caspase inhibitor, z-VAD, whereas selective inhibitors of other death pathways did not alter lethality (Figure 1E). Similar effects were observed following ⍺-amanitin treatment in U2OS cells (Figures 1E, S1H, and S1I). Furthermore, inhibiting apoptosis suppressed the lethality of transcriptional inhibitors in a panel of genetically unrelated cell lines (Figures 1E, S1J, and S1K; Table S1). These data reveal that transcriptional inhibition—even complete and prolonged transcriptional inhibition—exclusively activates a single cell death pathway, the cell-intrinsic apoptotic response.

### Apoptosis is activated prior to passive loss of apoptotic regulatory proteins

RNA Pol II was completely inhibited at the doses we used, and as expected, RNA levels decreased following loss of RNA Pol II (Figures S1L–S1N). However, at the time of death initiation, the RNA pool was only modestly decreased, with the total abundance of mRNAs decayed by ∼25% (Figures S1O and S1P). Furthermore, at the protein level, we observe no change in expression of canonical apoptotic regulatory proteins 4–8 h after triptolide exposure (Figure S2A). Thus, rapid initiation of apoptosis following loss of RNA Pol II could not be easily explained by the passive loss of mRNA or downstream decrease in protein expression.

Cellular behaviors did not appear to be dysregulated, even following a more substantial loss of RNAs. For instance, we observed similar RNA decay rates in U2OS and U2OS-BAX/BAK DKO cells (Figure S2B; Table S2). However, 4 days following triptolide exposure—when U2OS were completely dead—BAX/BAK DKO cells displayed no overt signs of dysregulation, despite having lost more than 90% of the mRNA pool (Figures 1F–1H and S2B–S2E). Even 4 days after triptolide exposure, BAX/BAK DKO cells were capable of re-adhering to plates following trypsinization, produced normal levels of ATP, and could activate DNA damage signaling (Figures S2F–S2K). Normal behaviors were maintained in BAX/BAK DKO cells through ∼10 days of triptolide exposure, after which cells began dying, with the entire population dying by 24 days (Figures 1G and S2L). Notably, passive death observed in BAX/BAK DKO cells was coincident with complete loss of all mRNAs, evaluated using absolute mRNA quantification (Figure 1H; Table S2). These data reveal that accidental cell death following loss of mRNA does indeed occur but is temporally distinct from the rapid death caused by RNA Pol II loss in cells that are proficient for activating apoptosis.

One potential explanation for these results is that lethality induced by triptolide and ⍺-amanitin stems from an off-target mechanism, unrelated to RNA Pol II. To address this, we evaluated the effects of RNA Pol II degradation in HAP1 cells, in which the endogenous RPB1 gene (*POL2RA*) is tagged with an auxin-inducible degron (HAP1-RPB1-AID) (Figure 1I, top).^19^ While auxin had no effect on wild-type (WT) HAP1 cells, HAP1-RPB1-AID cells displayed rapid cell death that initiated shortly after RNA Pol II degradation (Figure 1I). Furthermore, lethality of Rpb1 degradation could be potently suppressed by co-treatment with z-VAD, consistent with the results observed for triptolide and ⍺-amanitin (Figure 1I). Taken together, these data reveal that apoptosis following loss of RNA Pol II occurs in response to an active process that is distinct from the effects of global loss of mRNA.

### Loss of hypophosphorylated RNA Pol II—but not loss of RNA Pol II activity—correlates with initiation of apoptosis

While apoptotic-proficient cells die before experiencing global loss of mRNA, these cells still lose short-lived transcripts (Figure S2C). Mcl-1 is a potent negative regulator of apoptosis, unique among apoptotic regulators in that it is rapidly turned over at both the mRNA and protein levels.^20–23^ Indeed, Mcl-1 is rapidly lost following transcriptional inhibition (Figures S3A and S3B). However, U2OS cells were insensitive to chemical inhibition or genetic knockout of *MCL1*, and loss of *MCL1* did not alter sensitivity to triptolide (Figures S3C and S3D).

**Figure 2.**
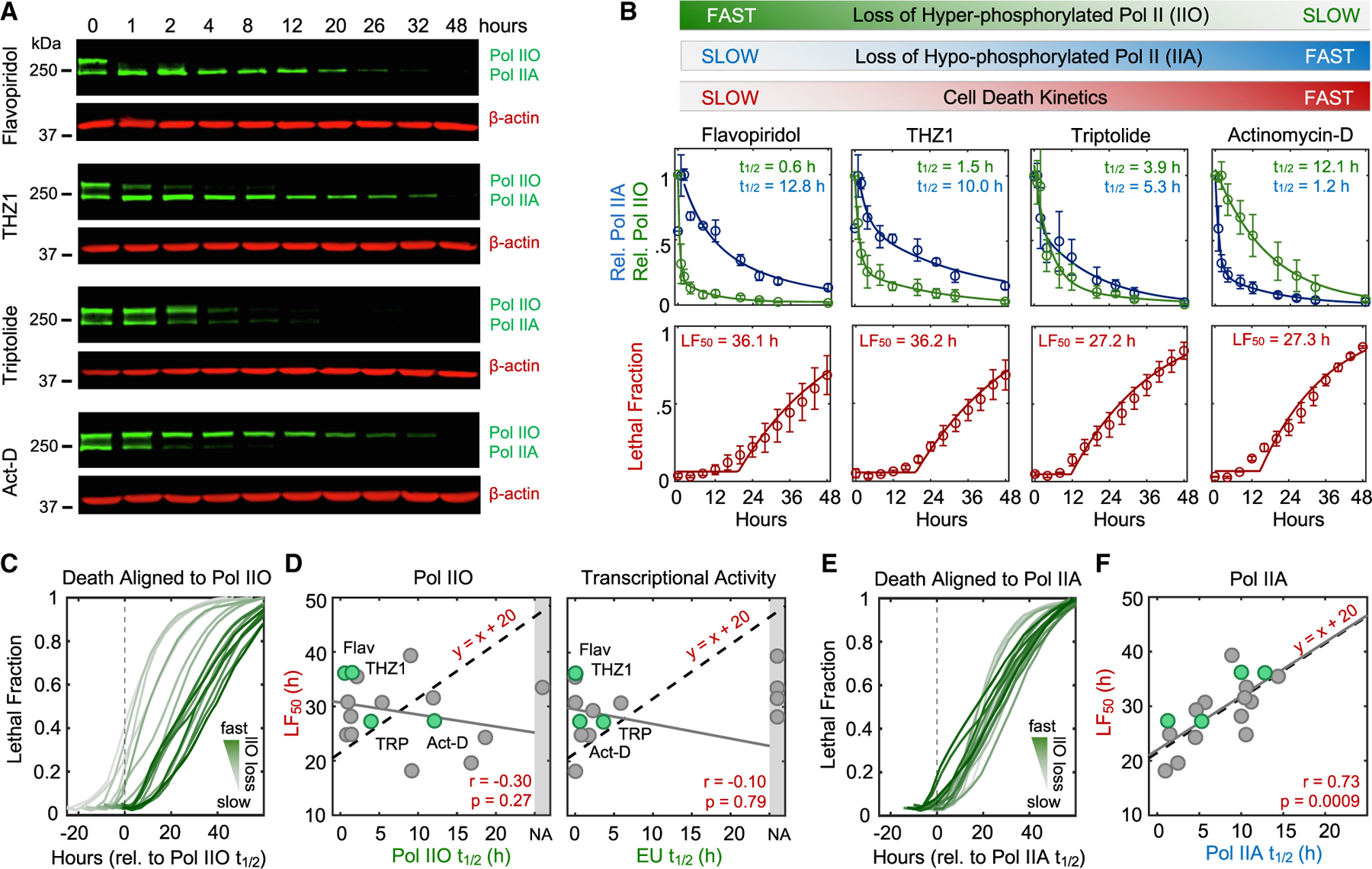
Loss of hypophosphorylated RNA Pol II correlates with onset of apoptotic death (A) Immunoblots of Rpb1 following flavopiridol (10 μM), THZ1 (1 μM), TRP (0.1 μM), or actinomycin-D (Act-D, 0.1 μM). Blots representative of 3 biological replicates. (B) (Top) Quantification of decay kinetics for RNA Pol IIO (hyperphosphorylated) and RNA Pol IIA (hypophosphorylated) from immunoblots in (A). t_1/2_ denotes time to 50% decay. (Bottom) Cell death kinetics. LF_50_ denotes time to 50% max observed death. (C and D) Relationship between loss of RNA Pol IIO, or loss of transcriptional activity, and cell death. (C) Cell death kinetics for 17 unique drug-dose combinations, aligned to t_1/2_ for loss of RNA Pol IIO (dashed vertical line). Mean of 3 biological replicates shown. (D) Correlation between LF_50_ and t_1/2_ for loss of RNA Pol IIO (left) or t_1/2_ for loss of transcriptional activity measured using EU incorporation (right). Solid gray line denotes fit to a linear regression model. Black dashed line denotes direct x = y relationship, shifted by a constant to account for a lag time between drug activity and cell death. (E and F) Relationship between loss of RNA Pol IIA and cell death. (E) Same as (C), except aligned to t_1/2_ for loss of RNA Pol IIA (dashed vertical line). (F) Correlation between t_1/2_ for loss of RNA Pol IIA and LF_50_. For panels with error bars, data are mean ± SD of 3 biological replicates. See also Figure S3 and Table S3.

We next sought to more generally test if loss of short-lived mRNAs following transcriptional inhibition causes apoptosis. One simple prediction of this model is that drugs inhibiting productive elongation faster will initiate death proportionally faster. To capture variation in RNA Pol II inhibition kinetics, we profiled transcriptional inhibitors that function with different inhibition timing: flavopiridol, THZ1, triptolide, actinomycin-D, α-amanitin, and ethynylcytidine.^24^ To quantify the kinetics of RNA Pol II inhibition, we first used immunoblotting of Rpb1 to distinguish between the levels of hyper- and hypo-phosphorylated RNA Pol II (Figure 2A). The upper band, called RNA Pol IIO, migrates slowly due to hyper-phosphorylation of the carboxyl-terminal domain (CTD).^25^ RNA Pol IIO is the productively elongating pool of RNA Pol II.^26^ The lower band, called Pol IIA, represents all other forms of RNA Pol II that are not productively elongating, including preinitiation complex (PIC) bound, promoter paused, early pause released, and free RNA Pol II.^27^ We observed significant variation in the timing of RNA Pol IIO loss, with some drugs, such as the Cdk9 inhibitor flavopiridol, causing IIO loss within minutes, while other inhibitors required several hours to completely lose RNA Pol IIO (Figure 2B; Table S3). However, drugs inducing the fastest loss of IIO were not particularly fast at activating cell death. The two fastest inhibitors, flavopiridol and THZ1, were, in fact, the two slowest activators of cell death (Figure 2B; Table S3). Overall, we found no correlation between the timing of cell death and the timing of RNA Pol IIO loss (Figures 2C and 2D; Table S3).

While RNA Pol IIO is the hyperphosphorylated form of RNA Pol II, it is not a precise measure of RNA Pol II transcriptional activity, particularly following transcriptional perturbations. Thus, we also used EU incorporation into nascent RNAs to explore the relationship between the timing of transcriptional inhibition and the timing of cell death (Figure S3E). No correlation was observed when comparing the timing of cell death to transcriptional inhibition quantified using EU incorporation (Figures 2D and S3F). These data suggest that death induced by RNA Pol II inhibition does not result from loss of short-lived mRNAs.

The effects of transcriptional inhibitors could be phenocopied using auxin-inducible degradation of Rpb1 (Figure 1I). Thus, we considered other mechanisms by which loss of RNA Pol II could activate death. While previous studies have generally focused on how transcriptional inhibitors affect RNA Pol IIO, our data highlight an underappreciated ability of these drugs to modulate hypophosphorylated RNA Pol IIA as well (Figure 2A). Notably, while loss of IIO was not correlated with activation of death, we observed a strong positive correlation between timing of cell death and timing of decay for RNA Pol IIA, the forms of RNA Pol II that are not actively elongating mRNA (Figures 2E, 2F, and S3G–S3I). Furthermore, the relationship between IIA decay and cell death was directly proportional, such that loss of RNA Pol IIA consistently preceded death by a fixed amount of time (Figure 2F). While only a correlative result, these data propose an alternative model whereby death following transcriptional inhibition is not related to inhibition of RNA Pol II activity and the subsequent loss of mRNA but instead may result directly from loss of the hypophosphorylated RNA Pol IIA protein.

### Loss of hypophosphorylated RNA Pol II initiates apoptosis

**Figure 3.**
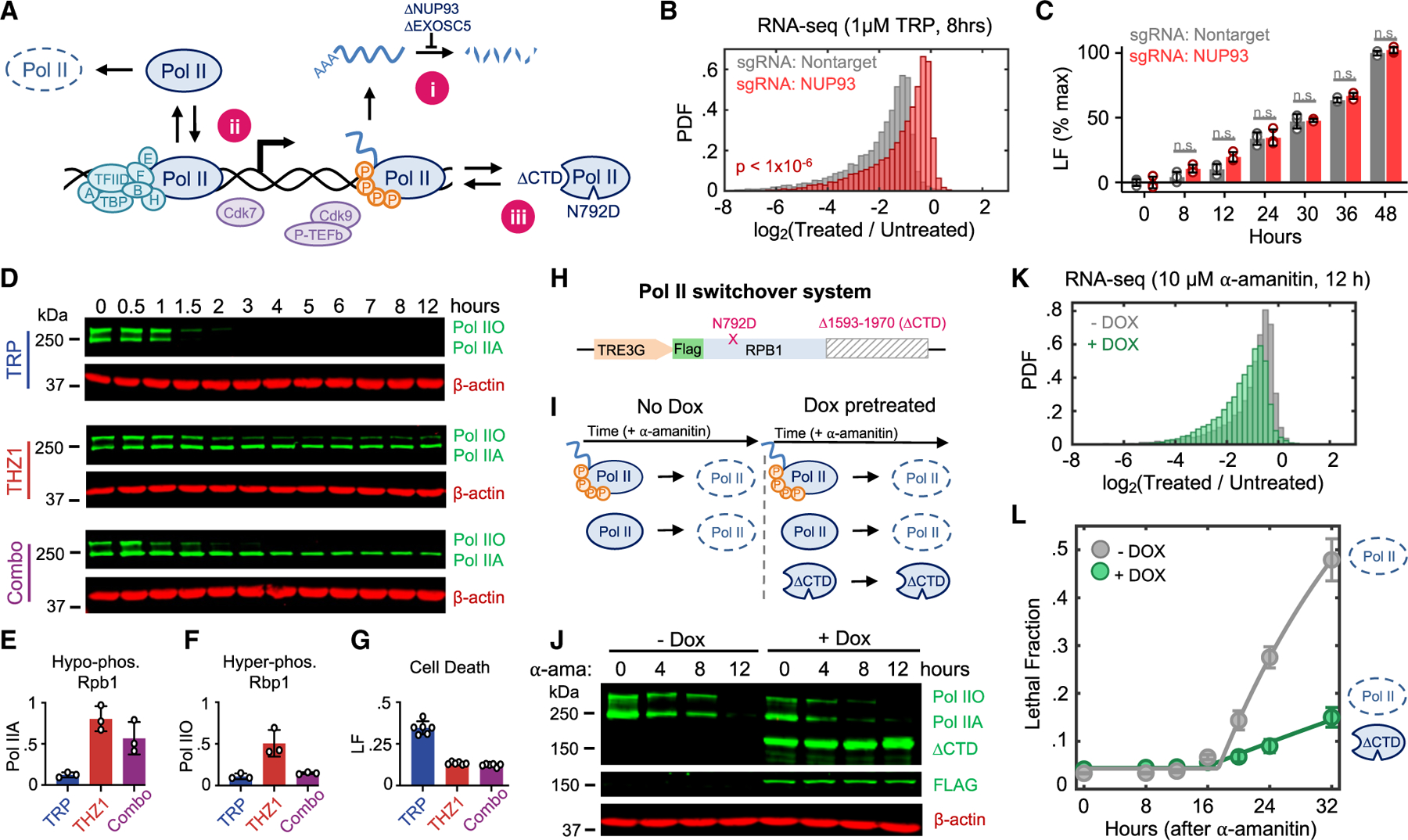
Loss of hypophosphorylated RNA Pol II initiates apoptosis (A) Schematic describing approaches for uncoupling RNA Pol II activity from protein levels (Ai–Aiii). (B) Absolute mRNA fold change following 1 μM TRP in U2OS expressing sgRNA-NUP93 or nontargeting sgRNA. Two-sided KS test *p* value shown. (C) LF for cells in (B) following TRP. Wilcoxon rank sum *p* value shown (n.s. *p* > 0.05). (D) Immunoblot of Rpb1 in U2OS following 1 μM TRP, 0.316 μM THZ1, or a combination of the two. Blots representative of 3 biological replicates. (E and F) Quantification of Rpb1 after 4-h drug exposure, associated with (D). (E) Hypophosphorylated (RNA Pol IIA). (F) Hyperphosphorylated (RNA Pol IIO). (G) LF at 24 h, measured in identical conditions to (D)–(F). Data are mean ± SD for 6 biological replicates. (H–L) RNA Pol II switchover system (H) diagram of RNA Pol II construct. (I) Experimental logic of switchover system. (J) Immunoblot of Rpb1 following 10 μM ⍺-amanitin. (K) Absolute mRNA fold changes following ⍺-amanitin. (L) LF following ⍺-amanitin. Mean ± SD for 5 biological replicates. Unless otherwise noted, error bars represent mean ± SD of 3 biological replicates. See also Figure S3 and Table S4.

A critical challenge for determining causality in this context is the mutual dependence between RNA Pol II protein and its enzymatic function: degrading RNA Pol II invariably inhibits RNA Pol II activity, and the existence of RNA Pol II protein relies on RNA Pol II-mediated transcription. To overcome this challenge, we developed three complementary strategies for uncoupling inhibition of RNA Pol II activity and the loss of RNA Pol II protein (Figure 3A). First, we sought to acutely degrade RNA Pol II without inducing subsequent loss of mRNAs by inhibiting mediators of RNA degradation (Figure 3Ai). NUP93 and EXOSC5 are critical mediators of mRNA concentration homeostasis, and acute knockdown of these proteins leads to an accumulation of mRNA.^7^ As expected, the mRNA pool in NUP93-KO cells was stabilized and significantly more resistant to triptolide-induced mRNA loss than control cells (Figure 3B; Table S4). Despite the stabilized mRNA pool, triptolide activated death to similar levels—and with similar kinetics—in control and NUP93-KO cells (Figure 3C). Similar results were observed in EXOSC5-KO cells (Figures S3J and S3K). Notably, some rapidly degraded transcripts—including MYC, WEE1, and JUN—continue to be rapidly degraded in NUP93-KO cells (Figure S3L). However, knocking out these genes did not kill U2OS cells, nor did knocking out these genes exacerbate triptolide-induced lethality (Figures S3M and S3N). These data again suggest that death following RNA Pol II inhibition cannot be explained by the passive loss of mRNA alone.

Second, we sought to buffer against triptolide-induced degradation of RNA Pol IIA while maintaining transcriptional inhibition (Figure 3Aii). Triptolide inhibits XPB, the helicase subunit of TFIIH.^11^ Triptolide-induced degradation of RNA Pol II requires Cdk7 activity, presumably because Cdk7 facilitates removal of the PIC and release of RNA Pol II from chromatin, enabling RNA Pol II degradation.^27^ Consistent with this, co-treatment with the Cdk7 inhibitor, THZ1, blocks triptolide-induced RNA Pol IIA degradation (Figures 3D and 3E). Importantly, THZ1 is itself a transcriptional inhibitor, and the combination of triptolide and THZ1 was as good at inducing RNA Pol IIO loss as either drug alone (Figures 3D and 3F). Despite the complete loss of hyperphosphorylated RNA Pol IIO, the addition of THZ1 suppressed the lethality of triptolide (Figures 3D and 3G). This paradox—in which the lethal effects of transcriptional inhibition can be suppressed by adding another transcriptional inhibitor—is in line with a model whereby lethality results from the degradation of hypophosphorylated RNA Pol II protein.

Finally, we asked if exogenous expression of a functionally inactive RNA Pol II mutant protein could rescue viability following degradation of endogenous RNA Pol II (Figure 3Aiii). To accomplish this, we designed an RNA Pol II “switchover” system (Figure 3H). This system features a doxycycline-inducible *RPB1* transgene completely devoid of the 52 heptapeptide repeat CTD.^28–31^ Truncated versions of the CTD can still initiate in some settings but have defects in splicing and termination, and a completely CTD-less Rbp1 cannot initiate transcription.^32,33^ In this CTD-less *RBP1* fragment, we also encoded an ⍺-amanitin resistance mutation, N792D, allowing the selective degradation of endogenous RNA Pol II.^12^ Conceptually, this system allows us to directly compare cell death outcomes, using isogenic cells that either have or do not have a pool of non-functional RNA Pol II protein (Figure 3I). As expected, ⍺-amanitin induced complete loss of endogenous RNA Pol II in both doxycycline-pretreated and untreated conditions (Figure 3J). However, only cells pretreated with doxycycline expressed a truncated, ⍺-amanitin-unresponsive Rpb1 protein (Figure 3J). Expression of Rpb1-N792D-ΔCTD did not inhibit ⍺-amanitin-induced loss of mRNA, validating that the CTD is required for transcription initiation (Figures 3K and S3O; Table S4). Importantly, expression of Rpb1-N792D-ΔCTD potently suppressed ⍺-amanitin-induced cell death (Figure 3L). Expression of Rpb1-N792D-ΔCTD had no effect on the lethality of unrelated apoptotic agents, and rendering Rpb1-ΔCTD sensitive to ⍺-amanitin abolished its ability to suppress death (Figures S3P and S3Q). These experiments demonstrate that cell death following transcriptional inhibition is caused by degradation of the hypophosphorylated form of RNA Pol II and not loss of RNA Pol II transcriptional activity.

### Chemogenetic profiling identifies regulators of triptolide-induced cell death

**Figure 4.**
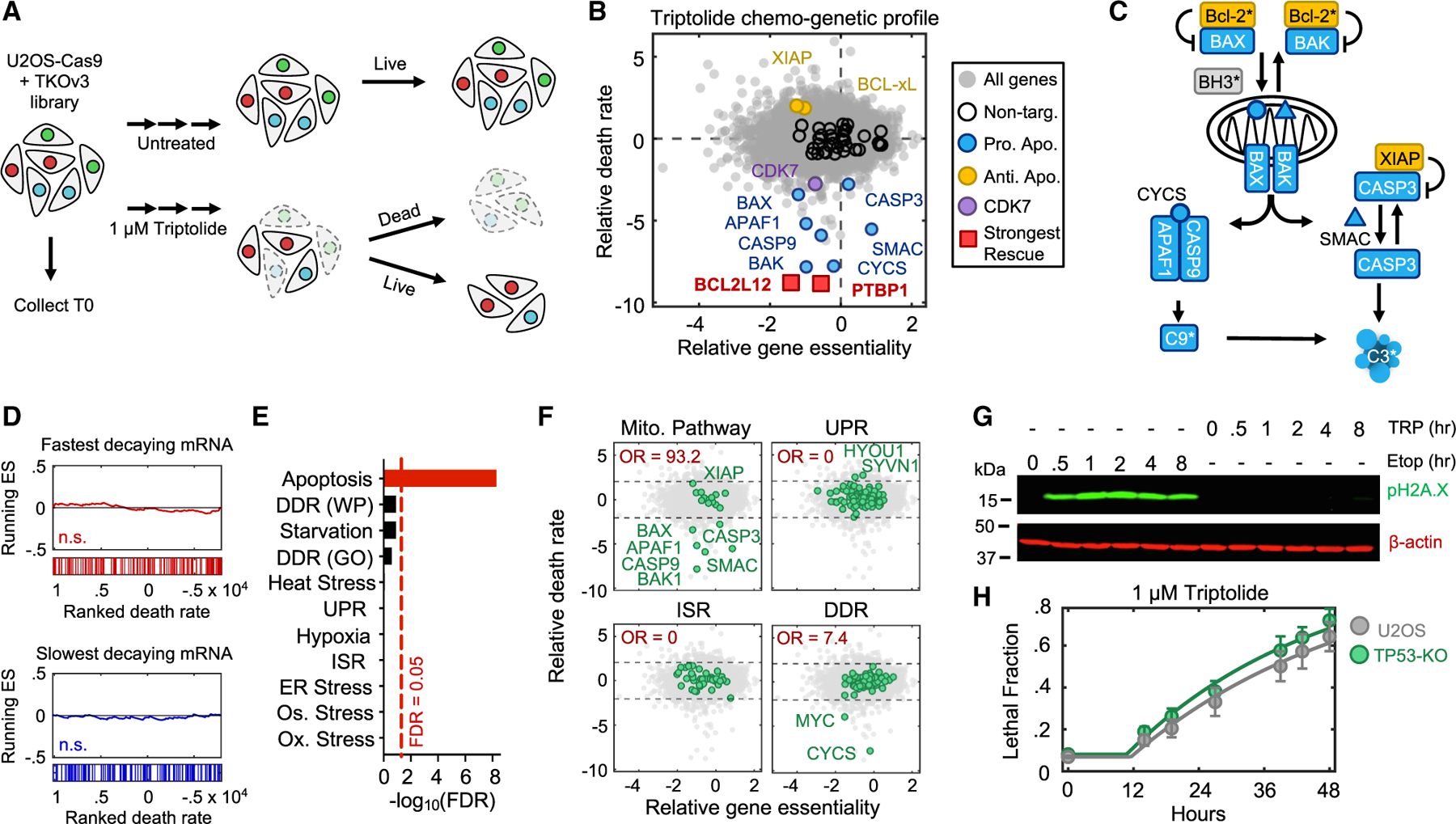
Genome-wide screen identifies regulators of TRP-induced apoptosis (A) Schematic of chemogenetic profiling to identify death regulatory genes. (B) Gene-level chemogenetic profiling data. Key death regulatory genes and nontargeting controls highlighted. (C) Simplified schematic of the cell-intrinsic apoptotic pathway. Bcl-2* denotes anti-apoptotic Bcl-2 family members. BH3* denotes pro-apoptotic BH3-only proteins. Regulators in blue/yellow were identified in chemogenetic profiling data. (D) Running enrichment scores (ESs) from gene set enrichment analysis on rank-ordered death rates, evaluating short-lived RNAs or long-lived RNAs. Nominal *p* value shown (n.s. *p* > 0.05). (E) Enrichment for stress response pathways among death regulatory genes identified by chemogenetic profiling. Significance was determined using a false discovery rate (FDR)-corrected one-tailed Fisher’s exact test. (F) Chemogenetic profiling data, highlighting genes associated with stress response pathways: Biocarta mitochondrial pathway (Mito. Pathway), unfolded protein response (UPR), integrated stress response (ISR), and DDR. Odds ratio for one-tailed Fisher’s exact test shown. Gray dashed lines: FDR = 0.1. (G) Immunoblot of phosphorylated-H2A.X(Ser139) following 31.6 μM etoposide or 1 μM TRP in U2OS. (H) LF following TRP in U2OS or U2OS-TP53-KO. Data are mean ± SD for 9 biological replicates. See also Figure S4 and Table S5.

RNA Pol II is a nuclear protein, but apoptosis is regulated at mitochondria. Thus, we next aimed to characterize how decreased levels of RNA Pol IIA are sensed and communicated to initiate apoptosis. Considering the lack of prior knowledge for which processes respond to loss of RNA Pol IIA, we evaluated this question using an unbiased genome-wide screening approach. We used Cas9-expressing U2OS cells to evaluate the effect of gene knockouts on triptolide-induced lethality.^34,35^ To identify genes that specifically regulate cell death, rather than proliferation, we developed a simple experimental approach to agitate plates, facilitating a mechanical separation of apoptotic corpses from live cells, such that both populations could be sequenced independently (Figures 4A and S4A–S4F).

Our screen identified more than 100 genes whose knockout reduced triptolide-induced cell death (Figures 4B and S4G; Table S5). Remarkably, this cohort included every gene encoding an essential pro-apoptotic regulatory protein (Figures 4B and 4C). We also identified key negative regulators of apoptosis, such as *XIAP* and BCL-xL (*BCL2L1*), which increased triptolide-induced lethality when deleted (Figures 4B and 4C). Furthermore, we identified triptolide-specific genetic dependencies, including *CDK7*, in line with our finding that THZ1 suppresses triptolide-induced death (Figures 3G and 4B). Importantly, regulators of triptolide-induced death were not enriched for genes encoding short half-life mRNAs, further highlighting the specificity of our screen and that mRNA decay does not drive cell death in this context (Figure 4D).

To clarify how RNA Pol II degradation activates apoptosis, we began by exploring if regulators of triptolide-induced death were enriched for genes involved in known stress response pathways. As expected, our screen was strongly enriched for apoptotic regulatory genes (Figure 4E). However, we did not identify any other stress response pathways that might facilitate activation of apoptosis (Figures 4E and 4F). Due to the proximity of RNA Pol II to DNA, we paid special attention to the DNA damage response (DDR). However, the DDR did not appear to be involved in triptolide-induced death: no regulators of DDR were hits in our screen, triptolide failed to activate DDR signaling, and knocking out TP53, an essential regulator of DDR-induced apoptosis,^36^ had no effect on triptolide-induced death (Figures 4F–4H).

### Apoptosis following RNA Pol II degradation requires PTBP1

**Figure 5.**
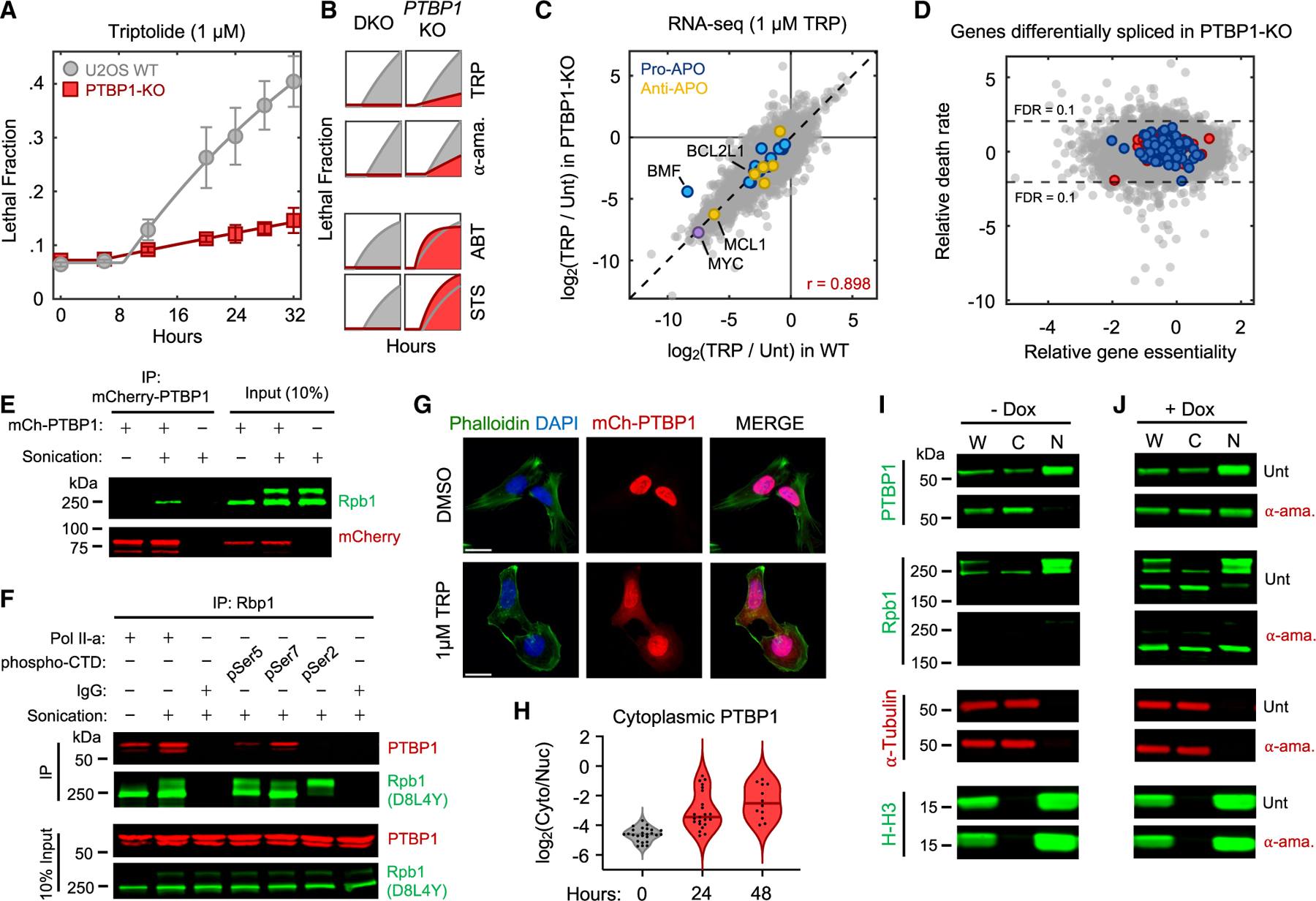
Apoptosis following RNA Pol II degradation depends on cytoplasmic translocation of PTBP1 (A) TRP-induced death in U2OS and PTBP1-KO. Data are mean ± SD for 6 biological replicates. (B) PTBP1-dependence for RNA Pol II degraders, or canonical apoptotic agents: 1 μM TRP, 10 μM ⍺-amanitin (⍺-ama.), 100 μM ABT-199 (ABT), and 0.5 μM staurosporine (STS). Drugs tested in U2OS, PTBP1-KO, or BAX/BAK DKO. LF kinetics with area under the curve colored: gray, WT; red, DKO or PTBP1-KO, as indicated. Areas based on mean of 6 biological replicates. (C) RNA-seq comparing drug-induced transcriptional changes in U2OS (WT) and PTBP1-KO, 8 h after TRP. Data normalized using ERCC spike-ins. Dashed line, x = y. Pearson correlation coefficient shown. Anti-apoptotic (anti-APO) and pro-apoptotic (pro-APO) regulators highlighted. (D) Chemogenetic profiling data, highlighting genes alternatively spliced in a TRP/PTBP1-KO dependent manner. Blue, increased in PTBP-KO; red, decreased in PTBP1-KO. See also Figure S5H. (E) CoIP of RNA Pol IIA with mCherry-PTBP1. (F) CoIP of PTBP1 with varied forms of RNA Pol II. (G) Localization of mCherry-PTBP1 ± TRP for 48 h. Scale bar, 25 μm. (H) Quantification of PTBP1 cytoplasm/nuclear ratio following TRP. (I and J) Fractionation to assess PTBP1 localization following exposure to ⍺-ama. W, whole-cell lysate; C, cytoplasmic; N, nuclear. (I) U2OS-RBP1-N792D/ΔCTD cells following 12-h 10 μM ⍺-amanitin. (J) As in (I), except with doxycycline (+Dox). See also Figures S5 and S6 and Table S6.

The gene whose deletion most strongly suppressed triptolide-induced death was not a core apoptotic effector, but PTBP1 (Figure 4B). PTBP1 is a multi-functional RNA-binding protein with well-established roles in alternative splicing and activation of internal ribosome entry segments (IRESs).^37–39^ We validated in U2OS cells that knocking out *PTBP1* suppressed triptolide-induced death, and re-expressing *PTBP1* in PTBP1-KO cells restored triptolide sensitivity (Figures 5A, S5A, and S5B). A similar phenotype was observed following exposure to α-amanitin and following *PTBP1* deletion in a panel of genetically diverse cell lines (Figures 5B and S5C). By contrast, canonical apoptotic agents, such as ABT-199 or staurosporine, were not affected by knocking out *PTBP1* (Figure 5B). Together, these data reveal that PTBP1 is required for apoptosis specifically in the context of RNA Pol II degradation.

Knocking out *PTBP1* suppressed lethality without altering RNA Pol II degradation, triptolide-induced loss of transcriptional activity, or triptolide-induced decay of mRNAs (Figures S5A–S5E). These data rule out the trivial explanation that loss of *PTBP1* inhibits triptolide activity. Furthermore, triptolide-induced changes in gene expression in U2OS and PTBP1-KO cells were nearly indistinguishable, further supporting our general finding that rapid loss of mRNA cannot explain the lethality of transcriptional inhibition (Figures 5C, S5E, and S5F).

We next explored mechanisms by which PTBP1 could regulate apoptosis following RNA Pol II degradation. Prior studies report a post-transcriptional function in which PTBP1 promotes tumor necrosis factor (TNF)-induced apoptosis by activating IRESs, facilitating translation of the apoptotic effector, APAF1.^40,41^ However, APAF1 protein expression was not altered during triptolide exposure, and APAF1 was expressed to similar levels in WT and PTBP1-KO cells (Figure S5F). PTBP1 is best described as a regulator of alternative splicing.^42–45^ Intriguingly, the influence of PTBP1 on triptolide-induced lethality also appears to be unrelated to its role in alternative splicing: other regulators of alternative splicing did not suppress triptolide-induced lethality, triptolide did not affect PTBP1-dependent splicing, triptolide-induced splicing changes were unaffected in PTBP1-KO cells, and differentially spliced genes in PTBP1-KO cells did not affect triptolide-induced death when knocked out (Figures 5D and S5G–S5I; Tables S5 and S6).^37^

To further explore how PTBP1 facilitates activation of apoptosis, we used immunoprecipitation to determine if PTBP1 interacts with RNA Pol II. Importantly, we found that only hypophosphorylated RNA Pol IIA co-immunoprecipitated with PTBP1 (Figure 5E). Reciprocal immunoprecipitations targeting specific post-translationally modified forms of Rbp1 confirm that PTBP1 does not interact with the productively elongating RNA Pol IIO but does interact with hypophosphorylated RNA Pol IIA (Figure 5F).

Many RNA-binding proteins localize to the nucleus and accumulate in the cytoplasm following transcriptional inhibition, though the reason for this is unclear.^46,47^ Curiously, triptolide-induced death was rescued by knocking out the nuclear exporter *XPO1*; conversely, knocking out nuclear importers, *RANGAP1* or *KPNB1*, exacerbated triptolide-induced lethality (Table S5). Thus, we next explored whether loss of RNA Pol IIA stimulated nuclear-to-cytoplasmic translocation of PTBP1. Using fluorescence microscopy and cell fractionation, we confirmed that PTBP1 is a nuclear protein in healthy cells (Figures 5G–5I). However, PTBP1 translocated to the cytoplasm following triptolide- or α-amanitin-induced RNA Pol II degradation (Figures 5G–5I, S5J, and S5K). Expression of CTD-less RNA Pol II blocked cytoplasmic translocation of PTBP1 and suppressed the lethality of α-amanitin (Figures 5J, S5J, and S5L). These data suggest that cytoplasmic trafficking of PTBP1 is critical for its death regulatory function.

We performed RNA sequencing (RNA-seq) on cytoplasmic and nuclear fractions in WT and PTBP1-KO cells. We found no evidence that altered localization of PTBP1 is associated with a change in mRNA localization that can causally explain triptolide-induced cell death: a cross-comparison of our RNA-seq and chemogenetic profiling datasets revealed no PTBP1-dependent changes to transcript expression or localization among genes that are required for triptolide-induced death (Figures S5M and S5N; Table S6). These data, again, support our observation that altered mRNA expression cannot causally explain cell death following transcriptional inhibition.

Given that PTBP1 binds to RNA Pol IIA in the nucleus, we presumed that PTBP1 translocation is an early event, upstream of key regulatory steps, such as mitochondrial outer membrane permeabilization (MOMP). Indeed, knocking out *PTBP1* blocked triptolide-induced activation of BAX and the release of cytochrome *c* from mitochondria (Figures S6A–S6C). These data confirm that PTBP1 translocation is an early event, upstream of MOMP. Taken together, these data support that PTBP1 is a genuine regulator of apoptosis, specifically following loss of RNA Pol IIA.

### BCL2L12 activates MOMP following RNA Pol II degradation and PTBP1 translocation

**Figure 6.**
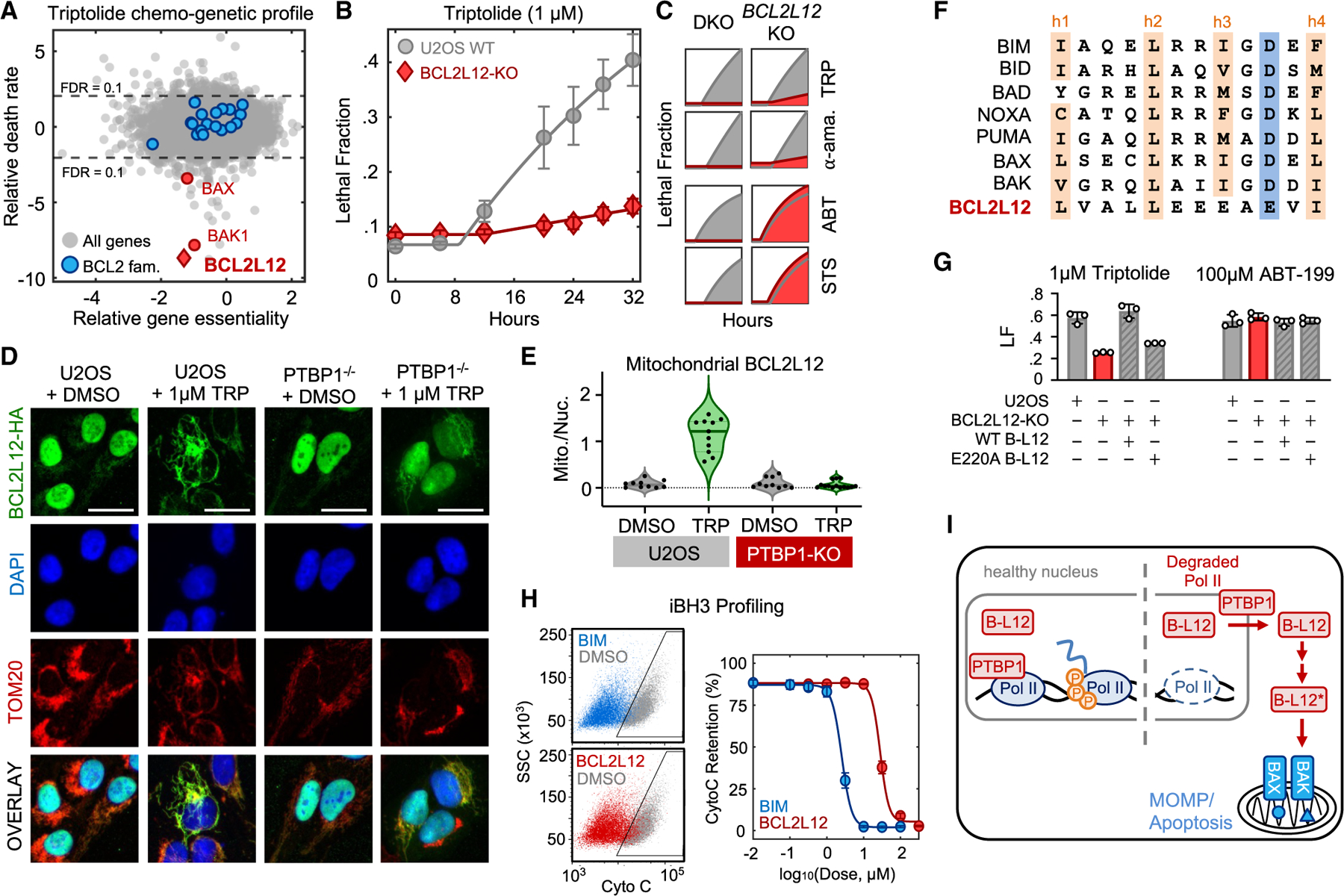
BCL2L12 activates apoptosis following PTBP1 translocation (A) Chemogenetic profiling data, highlighting BCL-2 family. (B) LF following TRP in U2OS and BCL2L12-KO. Data are mean ± SD of 6 biological replicates. (C) BCL2L12-dependence for RNA Pol II degraders or canonical apoptotic agents, as in Figure 5B. (D) Localization of HA-tagged BCL2L12 following TRP or vehicle for 24 h. Scale bar, 25 μm. (E) Quantification of BCL2L12 mitochondria:nuclear ratio, 24 h after TRP or vehicle. (F) Alignment of BH3 domains from established pro-apoptotic proteins and the BH3-like domain of BCL2L12. Conserved hydrophobic residues (h1–h4) and an invariant charged residue (blue) highlighted. (G) LF after the indicated compounds, in U2OS or BCL2L12-KO, expressing either BCL2L12 (WT B-L12) or mutant BCL2L12 (E220A B-L12). (H) iBH3 profiling of the BCL2L12 BH3-like domain. BIM is shown for comparison. (Left) Cytochrome *c* (CytoC) release in U2OS-BCL2L12-KO with 100 μM BIM-BH3, 100 μM BCL2L12-BH3-like peptide, or vehicle. (Right) iBH3 profiling across doses of BIM or BCL2L12 peptides. Data are mean ± SD of 3 biological replicates. (I) Schematic of PDAR. See also Figure S6.

We next aimed to address how apoptosis is activated following RNA Pol II degradation and PTBP1 translocation. Apoptosis is typically initiated by BH3 domain-containing proteins in the Bcl-2 family.^48,49^ Notably, our genetic screen identified every essential apoptotic effector downstream of MOMP; however, *BCL2L12* was the only Bcl-2 homology (BH) domain-containing gene that was required for triptolide-induced death, aside from BAX and BAK themselves (Figure 6A). BCL2L12 is a poorly understood protein that has previously been characterized as having an anti-apoptotic function.^50,51^ Nonetheless, we validated our genetic screening results, confirming that knocking out *BCL2L12* strongly suppresses triptolide-induced death in U2OS and other cell lines (Figures 6B, S6D, and S6E). *BCL2L12* was specifically required for the lethality of RNA Pol II degraders but not other apoptotic drugs (Figure 6C). Also, knocking out *BCL2L12* blocked triptolide-induced activation of BAX and release of cytochrome *c* (Figures S6F and S6G). These data confirm that, like PTBP1, BCL2L12 is required for activation of apoptosis downstream of RNA Pol II degradation and upstream of MOMP.

Notably, although many isoforms of BCL2L12 have been reported,^52^ we did not observe any PTBP1-dependent changes in splicing of BCL2L12 (Figures S5H, S5I, and S6H; Table S6). Thus, we next aimed to clarify the functional relationship between BCL2L12 and PTBP1. We began by exploring if PTBP1 translocation alters BCL2L12 localization. To visualize BCL2L12, we created an hemagglutinin (HA)-tagged BCL2L12, which was capable of restoring triptolide sensitivity in BCL2L12-KO cells (Figure S6I). Using fluorescence microscopy, we found that BCL2L12 has a predominately nuclear localization, although some staining was also observed at the mitochondria (Figure 6D). Following triptolide exposure, BCL2L12 lost nuclear localization and accumulated at mitochondria (Figures 6D, 6E, and S6J). Furthermore, triptolide-induced re-localization of BCL2L12 was blocked in PTBP1-KO cells (Figures 6D and 6E). Knocking out *BCL2L12* did not affect RNA Pol II degradation-induced translocation of PTBP1 (Figures S6K–S6M). These data suggest that PTBP1 is upstream of BCL2L12 and facilitates the release of BCL2L12 from the nucleus.

BCL2L12 is atypical within the Bcl2 family, as it contains only the BH2 homology domain, but not the BH1 or BH4 domains that typically exist in anti-apoptotic Bcl2 family proteins, or the BH3 domain that exists in all pro-apoptotic members of the Bcl2 family. Thus, it was unclear how BCL2L12 can function as an activator of apoptosis in the context of RNA Pol II degradation. However, prior studies note that BCL2L12 contains a non-canonical “BH3-like” domain.^53^ Given that BH3 domains are critical for the pro-apoptotic functions of apoptotic activators/sensitizers, we next determined if the BH3-like domain within BCL2L12 has pro-apoptotic activity. To address this, we mutated E220, a critical residue required for the function of other pro-apoptotic BH3 proteins (Figure 6F).^49,54^ BCL2L12-E220A failed to activate cell death following exposure to triptolide but did not affect the lethality of other apoptotic drugs (Figures 6G and S6N). These data suggest that the BH3-like domain of BCL2L12 is critical for its pro-apoptotic function.

To further characterize the pro-apoptotic function of BCL2L12, we used iBH3 profiling to test if the BH3-like peptide in BCL2L12 could induce MOMP.^55^ We found that the BCL2L12 BH3-like peptide induces MOMP with an EC_50_ of 20–30 μM, which was less potent than the strong activator BIM and in line with potencies of other BH3 proteins when tested in unprimed/untreated cells (Figures 6H and S6O).^56–58^ The activity of the BH3-like peptide was similar in WT and PTBP1-KO cells, consistent with our observation that BCL2L12 functions downstream of PTBP1 (Figure S6P). Taken together, these data reveal a basic framework for how apoptosis is activated upon RNA Pol II degradation. In healthy cells, PTBP1 and BCL2L12 are largely localized to the nucleus, and PTBP1 interacts with the hypophosphorylated forms of RNA Pol II. Upon loss of hypophosphorylated RNA Pol II, PTBP1 promotes the cytoplasmic translocation of BCL2L12, which initiates MOMP and cell death using the conventional intrinsic apoptotic pathway (Figure 6I).

### Multiple anti-cancer drugs owe their lethality to loss of RNA Pol IIA

**Figure 7.**
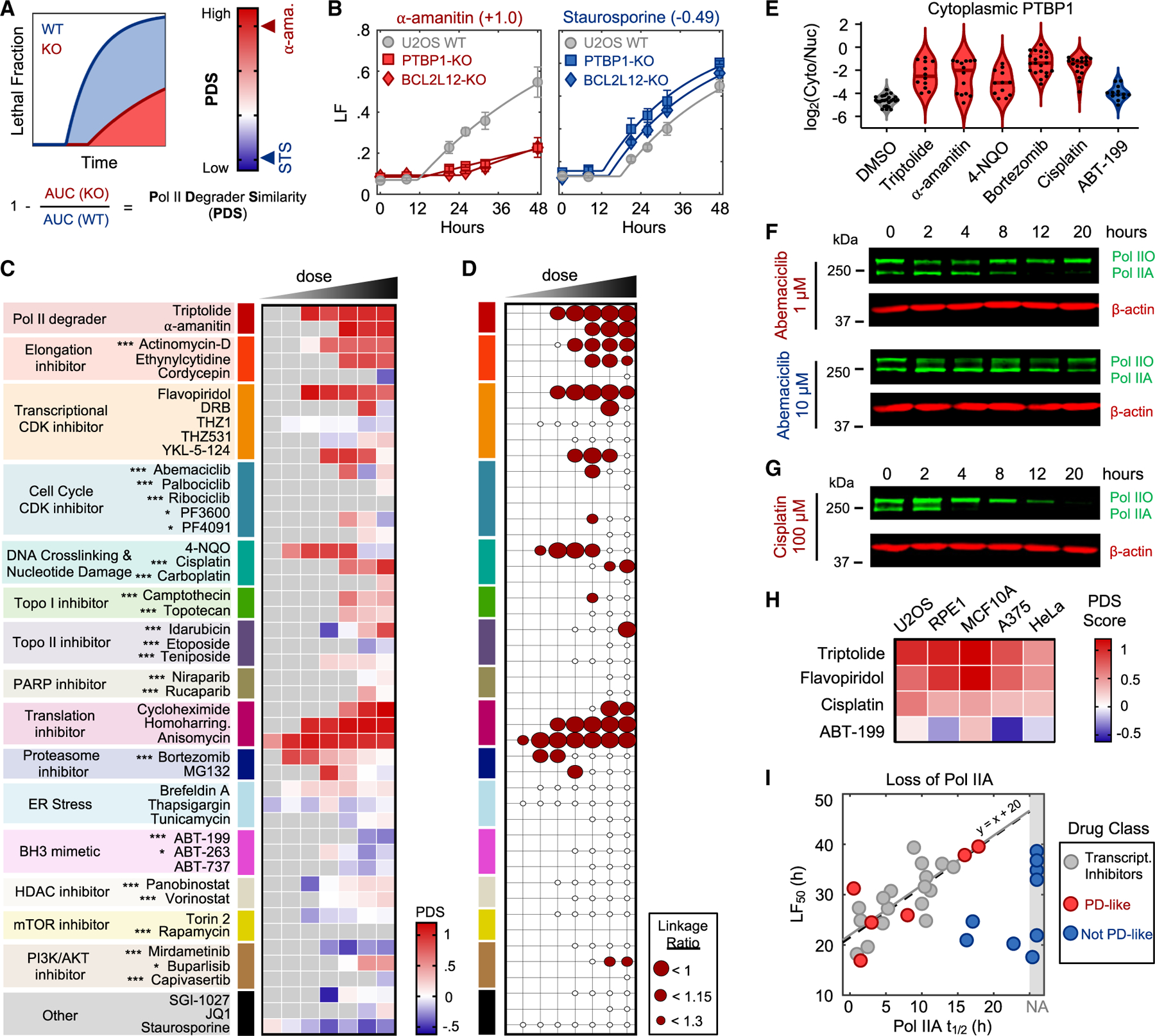
Commonly used anti-cancer drugs owe their lethality to RNA Pol II degradation (A) Strategy to identify PDAR-dependent drugs. KO refers to genetic dependencies observed for RNA Pol II degraders. (B) Cell death in U2OS for a high-scoring drug (10 μM ⍺-ama) and low-scoring drug (3.16 μM STS). Data are mean ± SD of 3 biological replicates. (C) Heatmap depicting PDS score across a 7-point, half-log dose range for 46 compounds. Data collected in U2OS. Gray boxes, non-lethal doses. ***Clinically approved; *clinically investigated. (D) Binary classifier, ordered as in (C). Red circles denote PD-like mechanisms. (E) mCherry-PTBP1 cytoplasmic-nuclear (Cyto/Nuc.) ratio following indicated compounds. (F) Immunoblot of Rpb1 in U2OS following 1 μM abemaciclib (PD-like) or a 10-fold higher dose (low PDS). (G) As in (F), but for cisplatin. (H) PDS across a panel of cell lines for 1 μM TRP, 10 μM flavopiridol, 100 μM cisplatin, and 100 μM ABT-199. (I) Relationship between LF_50_ and RNA Pol IIA t_1/2_. Data in gray denote established transcriptional inhibitors, as shown in Figure 2F. Red and blue data represent PD-like and non-PD-like compounds, respectively. See also Figure S7 and Table S7.

We next aimed to investigate if RNA Pol II degradation-dependent death could be valuable in a therapeutic context. To address this question, we tested commonly used lethal compounds—including clinically relevant drugs that are not conventionally considered transcriptional inhibitors—and determined if the lethality of these drugs depended on genes required for triptolide-induced death: *PTBP1*, *BCL2L12*, and *BAX*/*BAK1* DKO. We generated a simple “Pol II degrader similarity” (PDS) score, which quantifies the extent to which a drug shares the genetic dependency profile of triptolide (Figures 7A and 7B). We applied this signature-based strategy across 46 diverse compounds, covering 16 drug classes (Figure 7C; Table S7). Strikingly, a wide array of PDS scores were observed, with variation both across and within drug classes (Figure 7C). High PDS scores were observed for most drugs that directly target RNA Pol II or transcriptional Cdk proteins (Figure 7C). By contrast, drugs inducing proteotoxic stress, such as brefeldin A, thapsigargin, or tunicamycin, were quite lethal but featured low PDS scores, confirming that RNA Pol II degradation activates death in a manner that is distinct from general dysregulation of protein levels (Table S7). Collectively, these data highlight that many drugs covering disparate mechanisms share the genetic dependencies observed for RNA Pol II degraders.

Each drug was evaluated across a range of doses, and we observed a large variation in PDS scores across doses of a drug. To more precisely categorize drug-dose pairs, we developed a binary classifier to evaluate our PDS data. We used a conservative probabilistic nearest neighbors-based machine-learning approach, in which a drug-dose pair was classified as “Pol II degradation-like” (PD-like) only if its proximity to validated RNA Pol II degrading drugs is within the neighborhood occupied by triptolide and α-amanitin (Figures S7A and S7B).^59,60^ Our classifier identified significant variation in drug classification across lethal doses of a drug (Figure 7D). For instance, idarubicin was lethal at the highest 4 tested doses but was only PD-like at the highest dose (Figure 7D). Conversely, 4-NQO was lethal at nearly all tested doses but, strikingly, only PD-like at intermediate doses (Figure 7D).

We next validated if key features of RNA Pol II degradation-dependent death are also observed for PD-like drugs. For some PD-like drugs, it appeared that the mechanism of activation must differ from what is observed for conventional transcriptional inhibitors. For instance, the proteasome inhibitor bortezomib inhibits RNA Pol II degradation; however, RNA Pol II should still be ubiquitinated and recruited to the proteasome^61^ and thus may no longer be available to sequester PTBP1. Thus, we first tested if PD-like compounds also cause PTBP1 translocation to the cytoplasm. Like triptolide, PD-like compounds 4-NQO, bortezomib, and cisplatin cause nuclear-to-cytoplasmic translocation of PTBP1 (Figure 7E).

We next sought to validate if PD-like drugs also cause loss of RNA Pol IIA, as information about RNA Pol IIO/A levels or RNA Pol II activity is not included in the classifier. We focused initially on drugs such as abemaciclib, which are classified as PD-like at an intermediate dose but, paradoxically, not at higher doses (Figure 7D). As predicted by our classifier, RNA Pol IIA is lost following exposure to 1 μM abemaciclib but not when exposed to a 10-fold higher dose (Figure 7F). Similar paradoxical dose-dependent loss of RNA Pol IIA was observed for other drugs, including the Cdk2/4/6 inhibitor PF3600 and idarubicin (Figures S7C–S7G). Thus, PD-like compounds also lose RNA Pol IIA, and this behavior was restricted to doses classified as PD-like.

Due to its widespread clinical use, we paid special attention to cisplatin, a chemotherapy used in the treatment of lung, ovarian, and testicular cancers, among others.^62^ Our classifier predicts a PD-like mechanism for cisplatin at all doses, causing high levels of death (Figure 7D). This was surprising, given that DNA damage can independently activate apoptosis through the DDR, which is observed for other drugs in this dataset. Consistent with our classifier, however, we observe RNA Pol IIA loss following cisplatin exposure at all doses inducing substantial lethality (Figures 7G and S7H–S7J). Furthermore, cisplatin retains high PDS scores across several genetically unrelated cell types (Figure 7H). These data suggest that the lethality of several DNA-damaging agents may hinge upon an RNA Pol II degradation-dependent death in both a drug- and dose-dependent manner.

Compounds that create bulky DNA lesions, such as 4-NQO and cisplatin, actually stall and degrade elongating RNA Pol IIO, and RNA Pol IIA is not directly degraded but rather “lost” due to being converted to RNA Pol IIO following initiation.^63^ Thus, considering that some PD-like compounds preferentially target RNA Pol IIO, we explored if the lethality of PD-like compounds is still kinetically coupled to the loss of hypophosphorylated RNA Pol IIA. For PD-like drugs, cell death kinetics were also well correlated with the loss of RNA Pol IIA, with timing consistent with what we observe for canonical transcriptional inhibitors (Figure 7I). Cell death kinetics for negative control drugs showed no relationship, and no correlation was observed between cell death timing and loss of RNA Pol IIO, even for drugs that preferentially target RNA Pol IIO (Figure 7I; Table S7). Taken together, these data reveal that mechanisms of drug-induced lethality vary in non-trivial ways across doses, and more specifically, that loss of RNA Pol IIA is a common mechanism of lethality across many unrelated drug classes.

## DISCUSSION

The fact that transcriptional inhibition is lethal to cells is hardly surprising. However, a common assumption has been that loss of transcription is toxic due to passive decay of mRNA and protein. We show that death following transcriptional inhibition initiates rapidly, well before cells experience stress from loss of mRNA or protein. Furthermore, we demonstrate that death occurs in response to an active signaling process, which is initiated by loss of the hypophosphorylated (i.e., not actively transcribing) form of RNA Pol II, not loss of RNA Pol II transcriptional activity. We also identify the critical events that signal loss of RNA Pol IIA to initiate apoptosis, specifically PTBP1/BCL2L12 translocation from the nucleus to the cytoplasm and BCL2L12-dependent activation of MOMP. Therefore, we propose that this process be termed the Pol II degradation-dependent apoptotic response (PDAR).

Why has this phenotype not been observed previously? Notably, the central issue is simply related to analytical resolution. Specifically, while apoptotic-deficient cells remain 100% viable following RNA Pol II degradation, these cells also cannot proliferate. Common drug response metrics misinterpret the level of lethality in this context because a non-proliferative drug-treated population is already very small compared with a rapidly dividing untreated population (Figure S1G).^64^ Furthermore, given the lack of mechanistic insight provided by conventional analysis methods, any observed difference could reasonably be interpreted as evidence that apoptosis makes a partial/negligible contribution to the lethality of transcriptional inhibition (Figure S1G). Our evaluations of transcriptional inhibitors used the growth rate-adjusted for death (GRADE) method,^65^ and mechanistic clarity provided by GRADE revealed that apoptotic-deficient cells completely arrest cell proliferation without activating any cell death (Figure S1F).

Prior to this discovery, substantial attention was focused on targeting transcription in cancer treatment. However, mechanisms promoting selective killing of cancer cells remained unclear and were generally presumed to be dependent on loss of rapidly turned-over proteins, such as Myc or Mcl-1.^66^ Our study highlights that many of these drugs owe their lethality to activation of PDAR. This begs the question, *why are cancer cells uniquely vulnerable to transcriptional inhibitors*? While our study does not answer this question, possibilities may include altered RNA Pol II dynamics,^67,68^ which may enable more flexible gene expression while sensitizing cells to PDAR. Alternatively, it is well-described that cancer cells are closer than normal cells to the threshold of apoptotic activation, also called mitochondrial priming.^69,70^ Given that transcriptional inhibition exclusively activates mitochondrially regulated apoptosis, the selectivity of transcriptional inhibition may also stem from differences in mitochondrial priming.

Notably, the definitions we used for classifying PDAR-dependent cell death are conservative, as a drug was only characterized as PDAR-dependent if no alternative mechanisms exist to facilitate death in the absence of this pathway. Our study also highlights that selective killing is possible for PDAR-dependent drugs, as we identified several conventional DNA-damaging chemotherapies, which have long-standing clinical utility, that also score as PDAR-dependent using our conservative criteria. Thus, future studies of PDAR are likely to reveal new biomarkers that improve our ability to use a wide array of anti-cancer drugs and also may reveal new ways to selectively kill cancer cells.

### Limitations of the study

Several mechanistic details of the PDAR pathway remain unclear. For instance, we did not clarify how much of the nuclear PTBP1 pool translocates to the cytoplasm upon loss of RNA Pol IIA, nor the amount that is required to propagate the PDAR signal. Additionally, it remains unclear how PTBP1 facilitates mitochondrial targeting of BCL2L12. Furthermore, because we lacked reagents for studying endogenous BCL2L12, our understanding of BCL2L12 localization was generated using an exogenous expression construct, and we were unable to evaluate how its level of expression and localization compare to the endogenous protein. Deeper mechanistic studies on endogenous BCL2L12 will help to understand the abundance of BCL2L12 in the nucleus/mitochondria and the relative contributions of these two pools. Our study demonstrates that loss of gene expression is not sufficient to activate apoptosis: knocking out PTBP1 or expressing a non-functional RNA Pol II rescued viability without altering the loss of gene expression. However, it is possible that altered gene expression cooperates with BCL2L12 to activate death by priming cells for apoptosis. The effect of transcriptional inhibition on priming was not explored in this study. Finally, we found that PDAR is the dominant form of death activated by many drugs. However, the extent to which PDAR contributes to the therapeutic efficacy of PD-like drugs has not been clarified. It remains possible that cell-autonomous killing is not required for the efficacy of some compounds or that mechanisms of lethality vary across cancer types or *in vivo*.

## RESOURCE AVAILABILITY

### Lead contact

Further information and requests for resources and reagents should be directed to the lead contact, Michael Lee (michael.lee@umassmed.edu).

### Materials availability

Requests for materials will be made available by the lead contact with a materials transfer agreement.

### Data and code availability

Custom scripts for curve fitting, LED kinetics, and drug GRADE are deposited on GitHub (https://github.com/MJLee-Lab). Custom scripts for other analyses are available upon request. RNA-seq data can be found in supplemental tables and as FASTQ files from the Gene Expression Omnibus (GEO) under accession numbers GEO: GSE283148 (triptolide time course), GEO: GSE283149 (NUP93-KO or EXOSC5-KO), GEO: GSE283150 (nuclear:cytoplasmic fractionation), and GEO: GSE283151 (RNA Pol II switchover system). Chemogenetic profiling data can be found as FASTQ files (GEO: GSE283147) and in Table S5. All other data are available in the main text or supplemental information.

## STAR★METHODS

### EXPERIMENTAL MODEL AND STUDY PARTICIPANT DETAILS

#### Cell Lines Culturing Conditions

U2OS, MDA-MB-157, A375, Hs578T, MDA-MB-453, MCF7, and HeLa cells were grown in DMEM (Corning, Cat# 10-017-CV) supplemented with 2mM glutamine (Corning, Cat# 25-005-CI). HT-29 cells were grown in McCoy’s 5A medium (Corning, Cat# 10-050-CV). WI-38 cells were grown in MEM medium (Thermo Fisher Scientific, Cat# 10370088) supplemented with 2mM glutamine. H1650 and PC9 cells were grown in RPMI medium (Thermo Fisher Scientific, Cat# 11875119). RPE-1 cells were grown in low glucose DMEM (Gibco, Cat# 11885-092) and Hams F-12 (Thermo Fisher Scientific, Cat# 11765054) mixed at a 1:1 ratio. HAP1 cells were grown in IMDM medium (Thermo Fisher Scientific, Cat# 12440053). MCF10A cells were grown in DMEM-F12 medium (Thermo Fisher Scientific, Cat# 11320033) supplemented with 5% horse serum (Thermo Fisher Scientific, Cat# 26050088), 20 ng/ml epidermal growth factor (EGF), 0.5 μg/ml hydrocortisone, 100 ng/ml cholera toxin (Sigma-Aldrich, Cat# C8052-2MG), 10 μg/ml insulin (Life Technologies, Cat# 12585014), and penicillin-streptomycin (Corning, Cat# 30-002-CI). Each media (expect that for MCF10A) was supplemented with 10% FBS (Peak Serum, Cat# PS-FB2, lot no. 21E1202) and penicillin-streptomycin (Corning, Cat# 30-002-CI). Hs578T cells were supplemented with 10μg/mL insulin (Thermo Fisher Scientific, Cat# 12585014). Cell lines were cultured in incubators at 37C with 5% CO2. Cell lines were maintained at low (<25) passage number from the original vial. U2OS, HT-29, WI-38, Hs578T, MDA-MB-453, MCF7, MDA-MB-157, A375, RPE-1, MCF10A and HeLa cells lines originated from female patients. H1650, PC9, HAP1 and HAP1-RPB1-AID cells originated from male patients. A375 cells were a gift from the Green laboratory (UMass Chan Medical School). HAP1 and HAP1-RPB1-AID cells were a gift from the Dekker laboratory (UMass Chan Medical School). RPE-1 cells were a gift from the Pazour laboratory (UMass Chan Medical School). PC9 cells were a gift from the Pritchard laboratory (Penn State University). All other cell lines mentioned above were purchased from ATCC and were authenticated using STR profiling with their identity confirmed.

U2OS-BAX/BAK1 double knockout cells (DKO) were generated previously.^71^ U2OS cells expressing Nuc::mKate2 (U2OS-mKate2+) were generated previously.^71^ All mKate2+ cells were generated by lentiviral infection with NucLight Red Lentivirus (Sartorius, 4627), followed by FACS selection. HAP1-RPB1-AID cells were treated with 450 μg/mL hygromycin for one week prior to use. U2OS-Cas9 expressing and U2OS-TP53 knockout cells were generated previously.^36^

#### Cell Line Engineering

##### U2OS-BAX/BAK-DKO-mKate2+ cells

All mKate2+ cells were generated by lentiviral infection with NucLight Red Lentivirus in U2OS-BAX/BAK-DKO cells, followed by FACS selection.

##### U2OS-PTBP1 and -BCL2L12 clonal knockout cells

U2OS-PTBP1 and U2OS-BCL2L12 clonal knockout cells were generated using CRISPR. For each gene, the highest-scoring sgRNA (see Table S1) was selected from the TKOv3 library and cloned into the pX330-puro plasmid using the single-step digestion-ligation protocol from the Zhang laboratory (available on the Zhang laboratory Addgene page). U2OS cells were then transiently transfected with the respective pX330-sgRNA-puro constructs using the FuGENE HD Transfection Reagent. Transfected cells were then selected with 1 μg/mL puromycin for 3 days, followed by replating and recovery for 2 days. Single cell clones were generated using limiting dilution. Clones were validated by sequencing and TIDE analysis,^72^ and for PTBP1 knockouts, immunoblotting.

#### Pooled single gene knockout cells using px330

U2OS-NUP93, -EXOSC5, -MCL1, -MYC, -WEE1, -TICRR, -JUN, -FBXO5, -DTL, -DBR1, -BCL2A1, and -NONT (nontargeting) knockout cells were generated using a transient transfection strategy. Briefly, sgRNA (see Table S1) were cloned into pX330-puro as described above. All constructs were sequence confirmed through Genewiz. U2OS cells were plated in six-well plates (300,000 cells per well) and left to adhere overnight. The following day, cells were transiently transfected with 2 μg of respective pX330-sgRNA-puro construct using FuGene HD Transfection Reagent. Cells were then selected using 1 μg/mL puromycin for either 2 days (for NUP93 and EXOSC5) or 3 days (for all others), followed by replating and expansion for either 1 day (for NUP93 and EXOSC5) or 2 days (for all others) prior to seeding for subsequent assays.

#### Knockout cells using the in4mer Cas12a system

To determine the genetic dependence of cell death on BAX, BAK1, PTBP1, and BCL2L12 across a panel of normal and cancer cell lines, the Cas12a in4mer system was used, as described previously.^82^ The in4mer systems allows for 4 independent guide RNAs to be co-infected with EnAsCas12a, allowing for both single- and multi-gene knockouts. Constructs were designed to target PTBP1 (with four guide RNAs), BCL2L12 (with four guide RNAs), or BAX and BAK1 (with two guide RNAs per gene). An intergenic-targeting construct (with four guide RNAs) was used as a control. Guide RNAs from the Inzolia library were designed into gBlocks (see Table S1) and synthesized through Integrated DNA Technologies. gBlocks were then individually cloned into the pRDA-550 vector (Addgene, Cat# 203398) using the NEBridge Golden Gate Assembly Kit (BsmBI-v2). All constructs were sequence confirmed through Genewiz and packaged into virus using Lenti-X 293T cells. Cell lines (U2OS, RPE-1, MCF10A, A375 and HeLa) were infected with virus and selected with puromycin.

#### RPB1 switchover cells

For the RPB1 switchover system, a FLAG-RPB1-N792D-ΔCTD fragment was PCR amplified (primers in key resources table) from FLAG-Pol2-WT (Addgene, Cat# 35175).^83^ The PCR fragment resulted in a RPB1 mutant that lacked the entire C-terminal domain (repeats 1–52) and included an N-terminal FLAG tag, an ⍺-amanitin resistance mutation, and homology regions to facilitate insertion into pLVX-Tre3G-IRES—a lentiviral vector for doxycycline-inducible gene expression. pLVX-Tre3G-IRES (Clontech, Cat# 631362) was digested with BamHI and NotI, gel extracted using the QIAquick Gel Extraction kit and purified. The PCR product was then inserted into the MCS1 of pLVX-Tre3G-IRES using Gibson Assembly. The resulting plasmid, pLVX-Tre3G-FLAG-RPB1-N792D-ΔCTD, was packaged into virus using Lenti-X 293T cells, along with a vector expressing the Tet-On 3G transactivator protein, pLVX-Tet3G. U2OS cells were infected with pLVX-Tet3G lentivirus, followed by selection with 600 μg/mL G418. Tet-3G expressing cells were then infected with pLVX-Tre3G-FLAG-RPB1-N792D-ΔCTD virus, and selected with 1 μg/mL puromycin. Single cell clones were generated using limiting dilution. Clones were selected for transgene expression levels approximating endogenous Rpb1 levels, determined using immunoblotting.

#### mCherry-PTBP1 expressing cells

The PTBP1 ORF was obtained as a Gateway entry clone in the pDONR223 vector (DNASU Clone ID: 513885). The ORF was transferred into a lentiviral destination vector, pHAGE-nDEST-mCherry^84^ using the Gateway LR Clonase II Enzyme Mix. The resulting plasmid, pHAGE-mCherry-PTBP1-IRES-PURO was sequence confirmed and packaged into lentivirus using Lenti-X 293T cells. U2OS-PTBP1-KO cells were infected with pHAGE-mCherry-PTBP1-IRES-PURO virus, and FACS sorted based on mCherry expression. Cells with exogenous Ptbp1 protein expression levels approximating that of wild-type cells, determined by immunoblotting, were used for further experiments. The resulting cells express exogenous PTBP1 with a N-terminal mCherry tag from a CMV promoter.

#### BCL2L12-Flag-HA expressing cells

The full length BCL2L12 ORF (334 amino acids) was obtained from the human ORFeome collection and provided as a Gateway entry clone in the pDONR223.^85^ The ORF was then transferred into a lentiviral destination vector, pHAGE-cDEST-FLAG-HA-IRES-PURO,^86^ using the Gateway LR Clonase II Enzyme Mix. The resulting plasmid, pHAGE-BCL2L12-FLAG-HA-IRES-PURO was sequence confirmed and packaged into lentivirus using Lenti-X 293T cells. U2OS-BCL2L12-KO or U2OS-PTBP1-KO cells were infected with pHAGE-BCL2L12-FLAG-HA-IRES-PURO virus and selected with 1 μg/mL puromycin. The resulting cells express exogenous BCL2L12 with a C-terminal Flag-HA tag.

To generate cells expressing BCL2L12 with a E220A mutation, the pHAGE-BCL2L12-FLAG-HA-IRES-PURO vector above was edited using the Q5 Site-Directed Mutagenesis kit with the primers: 5’ - GCTGGAGGAGgcgGCAGAAGTCA - 3’ and 5’- AGG GCCACCAGCCTC – 3’. The resulting plasmid, pHAGE-BCL2L12-E220A-FLAG-HA-IRES-PURO was sequence confirmed and packaged into lentivirus using Lenti-X 293T cells. U2OS-BCL2L12-KO cells were infected with pHAGE-BCL2L12-E220A-FLAG-HA-IRES-PURO virus and selected with 1 μg/mL puromycin.

### METHOD DETAILS

#### Immunoblotting

Cells were seeded in 6-well plates (300,000 cells per well) or 10-cm dishes (1.5 × 10^6^ cells per dish) and left to adhere overnight, unless otherwise stated. Cells were treated the next morning. At indicated timepoints, media were removed and collected. Samples were washed once with PBS, and the wash was added to the collected media. SDS-lysis buffer (50 mM Tris-HCl, 2% SDS, 5% glycerol, 5 mM EDTA, 1 mM NaF, 10 mM β-glycerophosphate, 1 mM PMSF, 1 mM Na_3_VO_4_, protease inhibitor tablet and phosphatase inhibitor tablet) was used to lyse the cells, along with any dead cells that were pelleted from the media and wash. Lysates were centrifuged through an AcroPrep 96 well 3.0 μm glass fiber/0.2 μm Bio-Inert membrane filter plate. Protein content was quantified with a BCA assay and normalized for equal loading. Samples were boiled for 5 minutes at 95C in 6x Laemmli buffer. Denatured samples were run on hand-poured SDS-PAGE gels. Gels were subsequently wet transferred onto nitrocellulose membranes and blocked in 1:1 PBS: Intercept Blocking Buffer for 1 hour at room temperature. Membranes were then incubated overnight on a rocking shaker at 4C in primary antibody diluted in 1:1 PBS-0.1% Tween: Intercept Blocking Buffer at the following dilutions: Anti-Rpb1-NTD rabbit mAb (clone D8L4Y), 1:1,000; Anti-DYKDDDDK Tag (FLAG) rabbit mAb, 1:1,000; Anti-Ptbp1 rabbit mAb, 1:1,000; Anti-Histone-H3 rabbit mAb, 1:2,000; Anti-⍺-tubulin mouse mAb, 1:10,000; Anti-pH2A.X (Ser139) rabbit mAb, 1:1,000; Anti-Mcl-1 rabbit mAb, 1:1,000; Anti-Apaf-1 rabbit mAb, 1:1,000; Anti-Bax rabbit mAb (clone D2E11), 1:1000; Anti-mCherry rabbit polyclonal Ab, 1:1000; Anti-HA Tag mouse mAb, 1:5000; Anti-c-Myc rabbit mAb, 1:1000. The following day, membranes were incubated in primary anti–β-actin antibody (diluted 1:15,000) for 1 hour at room temperature, followed by two 5-minute washes with PBS-0.1% Tween. Membranes were then incubated with secondary antibodies (diluted 1:15,000 in 1:1 PBS-0.1% Tween: Intercept Blocking Buffer) conjugated to infrared dyes (IRDye 680RD goat anti-mouse immunoglobulin G (IgG) secondary; IRDye 800CW goat anti-rabbit IgG secondary) for 1 hour at room temperature. Following four 5-minute washes with PBS-0.1% Tween and one 5-minute wash with PBS, blots were visualized using a LI-COR Odyssey CLx scanner. Raw protein expression levels were quantified in LI-COR’s ImageStudio software, background subtracted, divided by β-actin signals to normalize for loading differences, then normalized per gel to a reference sample (generally, an untreated sample). All immunoblots for Rpb1 use Anti-Rpb1-NTD rabbit mAb (clone D8L4Y), unless otherwise stated.

#### Immunoprecipitation and Co-immunoprecipitation

To measure activated Bax protein, an active conformation-specific Bax antibody (clone 6A7) was used.^87^ U2OS, U2OS-BCL2L12-KO, or U2OS-PTBP1-KO cells were plated onto 10cm plates and left to adhere overnight. The following day, cells were treated with 1 μM triptolide or vehicle, along with 50 μM z-VAD-FMK, for 24 hours. Cells were washed once with PBS, and lysed on the plate in 500 μL lysis buffer (10 mM HEPES pH 7.4, 150mM NaCl, 1% CHAPS, supplemented with protease and phosphatase inhibitor tablets). Replacement of 1% CHAPS with 1% Triton-X100 in the lysis buffer facilitates and active conformation of Bax and was used as a positive control. Cells were scraped in lysis buffer and pooled, then lysates were tumbled at 4C for 20 minutes. Lysates were then centrifuged at 16,100xg for 20 minutes at 4C. Supernatant was saved, and protein concentration was determined using the BCA assay. Samples were normalized to 450 μg protein in 550 μL, 50 μL was saved as input, then samples were incubated with 4 μg mouse anti-human Bax clone 6A7 (active Bax), or concentration matched mouse IgG1, κ isotype control, overnight at 4C. The following morning, samples were added to 25 μL pre-washed protein G magnetic beads and rotated for 2 hours at 4C. Complexes were then magnetically separated and washed 3 times in lysis buffer. Proteins were eluted from the beads by boiling samples in Laemmli buffer for 5 minutes at 95C. Equal volumes of IPs were separated by SDS-PAGE as described above, and total Bax was detected using anti-Bax (clone D2E11).

To identify protein-protein interactions between Ptbp1 and Rpb1, reciprocal Co-Ips were performed. To pull down Ptbp1, U2OS-PTBP1-KO cells reconstituted with mCherry-PTBP1 (U2OS-mCherry-PTBP1, detailed above) were lysed on ice in lysis buffer (10 mM Tris-HCl, 100 mM NaCl, 2.5 mM MgCl_2_, 0.5% NP-40, 1 mM PMSF, and protease inhibitor tablets). Wild-type U2OS cells were used as a control. Cells were scraped and collected in lysis buffer and incubated on ice for 30 minutes. Indicated samples were then sonicated using a Sonics VCX 130 Vibra Cell sonicator (10 seconds on and 10 seconds off, for 3 cycles at 30% amplitude). Lysates were centrifuged at 12,000 xg for 20 minutes at 4C. Supernatant was saved, and protein concentration was determined using the BCA assay. Samples were normalized to 1200 μg protein in 600 μL each, 100 μL was saved as input, then each sample was incubated with 25 μL of pre-washed RFP-Trap magnetic agarose beads and incubated at 4C for 2 hours with rotation. Beads were then washed 4 times with lysis buffer. Proteins were eluted from the beads by boiling samples in Laemmli buffer for 5 minutes at 95C. Equal volumes of IPs were separated by SDS-PAGE as described above.

For co-immunoprecipitation with Rpb1 in various phosphorylated states, U2OS cells were lysed on ice in lysis buffer (10 mM Tris-HCl, 100 mM NaCl, 2.5 mM MgCl_2_, 0.5% NP-40, 1 mM PMSF, and protease inhibitor tablets). Cells were scraped and collected in lysis buffer and incubated on ice for 30 minutes. Indicated samples were then sonicated using a Sonics VCX 130 Vibra Cell sonicator (10 seconds on and 10 seconds off, for 3 cycles at 30% amplitude). Lysates were centrifuged at 12,000 xg for 20 minutes at 4C. Supernatant was saved, and protein concentration was determined using the BCA assay. Samples were normalized to 1200 μg protein in 600 μL each and 100 μL was saved as input. To pull down hypo-phosphorylated Rpb1, samples were incubated with 8 μg of anti-Rpb1 mouse mAb clone 8WG16. To pull down Rpb1 phosphorylated at serine 5 of the CTD, samples were incubated with anti-phospho-Rpb1 CTD (ser5) Rabbit mAb at a 1:100 dilution. To pull down Rpb1 phosphorylated at serine 7 of the CTD, samples were incubated with anti-phospho-Rpb1 CTD (ser7) Rabbit mAb at a 1:100 dilution. To pull down Rpb1 phosphorylated at serine 2 of the CTD, samples were incubated with anti-phospho-Rpb1 CTD (ser2) Rabbit mAb at a 1:50 dilution. Samples were incubated for 4 hours at 4C with rotation, then added to 25 μL pre-washed protein G magnetic beads and rotated for 2 hours at 4C. Complexes were then magnetically separated and washed 4 times in lysis buffer. Proteins were eluted from the beads by boiling samples in Laemmli buffer for 5 minutes at 95C. Equal volumes of IPs were separated by SDS-PAGE as described above. To avoid interference with IgG heavy chain when blotting for PTBP1, the Quick Western Kit was used.

#### Proteome profiler analysis of apoptotic proteins

Proteome Profiler Apoptosis Arrays were performed according to the manufacturer’s instructions. Briefly, U2OS cells were collected at 1.5 million cells per condition and lysed according to instructions. An equal volume of lysate (250 μL) was loaded per array. Blot arrays were visualized using a LI-COR Odyssey CLx scanner, and protein expression was quantified in LI-COR’s ImageStudio software.

#### Quantification of ATP molecules per cell

To quantify ATP amount per cell following prolonged triptolide treatment, an approach was taken to measure ATP amount using CellTiter Glo normalized for cell number measured using the FLICK assay (see below for details). WT or DKO cells were plated at 15,000 cells per well in 96-well plates. The following day, cells were treated with 1 μM triptolide for 4 days. Untreated plates were also prepared such that all conditions could be processed at the same time. At assay endpoint, relative ATP amount was measured using CellTiter Glo, according to manufacturer’s recommendations. In parallel, relative cell number (both live and dead) was measured using FLICK. Absolute ATP amount was determined by generating an ATP standard curve. Similarly, absolute cell number was determined by generating a cell titration in FLICK. Thus, quantifying both cell number and total molar ATP amount enabled an average estimate of ATP molecules per cell.

#### Labelling and measurement of nascent transcription

Methods for labelling and quantification of nascent transcription were adapted from methods described previously.^88^ Cells treated with transcriptional inhibitors were labeled with 1 mM 5-ethynyl uridine for 1 hour in existing media. Cells were then washed with PBS, trypsinized, pelleted, and washed with cold PBS. Cells were then fixed in 4% fresh formaldehyde in PBS at room temperature for 15 minutes. Fixed cells were washed with cold PBS, pelleted, resuspended in ice-cold 100% methanol, and stored at −20C overnight. The following day, the methanol was removed, and the cells were washed twice with PBS-0.1% Tween. Cells were then incubated for 30 minutes at room temperature with 86.5 μL Click-iT reaction buffer, 4 μL CuSO_4_ buffer, 0.125 μL Alexa Fluor-azide-488 and 10.3 μL Click-iT reaction buffer additive (all from Thermo Fisher Scientific, C10329). Cells were washed once with 100 μL Click-iT reaction rinse buffer (from Thermo Fisher Scientific, C10329), then washed two more times with PBS-0.1% Tween. Samples were resuspended in PBS-0.1% Tween, filtered, and run on a Miltenyi MACSQuant VYB cytometer with laser and filter settings appropriate for reading Alexa-488. Data were analyzed using FlowJo software.

#### RNA sequencing

For evaluation of mRNA abundance following long-term triptolide exposure in DKO cells, DKO and WT cells were seeded onto six-well plates (300,000 cells per well) and left to adhere overnight. Cells were then drugged the next day (D0). To evaluate the transcriptome of WT cells after 1 day of triptolide, cells were cotreated with 50 μM z-VAD to inhibit loss of material from cell death. Due to the long assay length, plating and drugging was staggered for each condition, such that the assay endpoint for each sample occurred simultaneously. For analyses that defined the 50 shortest or 50 longest half-life RNAs, these lists of RNAs were based on prior studies.^89^

For evaluation of mRNA abundance in the context of NUP93 or EXOSC5 knockout, knockout and nontargeting cells were seeded onto six-well plates (300,000 cells per well) and left to adhere overnight. Cells were then drugged the next day.

For evaluation of mRNA abundance in Pol II switchover cells, Rpb1-N792D-ΔCTD expressing cells were seeded onto six-well plates (100,000 cells per well) and left to adhere overnight. The following day, the media was removed and replaced with fresh media with or without 2 μg/mL doxycycline. 48 hours later, drug or vehicle was gently added to the existing media.

For the extraction of mRNA for the experiments above, at assay endpoint cells were trypsinized, counted using a hemacytometer, pelleted, washed with PBS, and moved immediately to RNA extraction. Total RNA was extracted using the RNeasy Plus Mini Kit according to manufacturer protocol. To facilitate absolute RNA quantification, polyadenylated ERCC standards were diluted 1:100 in nuclease-free water, and added to each sample at 1 μL/100,000 cells. ERCC spike-ins were added immediately following lysis of cells in RLT-plus buffer, and prior to all subsequent total RNA extraction steps using the RNeasy Plus kit. All RNA integrity numbers (RIN) were greater than 9, as measured on a 4150 Tapestation System. mRNA was isolated using the NEBNext poly(A) mRNA Magnetic Isolation Module. RNAseq libraries were prepared with the NEBNext Ultra II Directional RNA Library Prep Kit using the NEBNext Multiplex Oligos for Illumina. Paired-end 2 × 50nt sequencing of libraries was performed in-house on the Illumina NextSeq2000 using the NextSeq 1000/2000 P^2^ XLEAP-SBS Reagent Kit.

#### Nuclear and cytoplasmic fractionation

Nuclear-cytoplasmic fractionation was performed in such a way to facilitate extraction of RNA and protein from the same cellular lysate. A similar protocol, excluding the RNA extraction steps, was used in fractionation experiments that only examined protein expression. Cells were plated at 6 million cells per condition (3 million cells per 15 cm plate) and left to adhere overnight. Cells were then drugged the next day. At assay endpoint cells were trypsinized, counted using a hemacytometer, pelleted, washed with PBS, and moved immediately to cellular fractionation. Cells were first resuspended in 900 μL hypotonic lysis buffer (50 mM HEPES pH 7.2, 150mM NaCl, 0.5 mM EDTA, 0.15% Triton X-100, 1 mM PMSF, protease inhibitor tablet, phosphatase inhibitor tablet, 20 U/mL SUPERase-In RNase inhibitor). Cells were pipet up and down 10 times, 1/3 of the lysate was taken as a whole-cell lysate (WCL) sample, and the cells were left to swell on ice for 10 minutes. Samples were centrifuged at 12,000xg for 30 seconds at 4C. The supernatant was collected as the cytoplasmic fraction (CYT). The nuclear pellet was washed once with 500 μL wash buffer (PBS containing 0.1% Triton X-100, 1mM EDTA and 20 U/mL SUPERase-In), centrifuged at 12,000xg for 30 seconds at 4C, and the wash buffer was completely removed. The nuclear pellet was then lysed in 400μL 1x nuclear lysis buffer (50 mM HEPES pH 7.2, 150mM NaCl, 0.5 mM EDTA, 1% Triton X-100, 0.5% Sodium Deoxycholate, 0.2% SDS, 1 mM PMSF, protease inhibitor tablet, phosphatase inhibitor tablet, 20 U/mL SUPERase-In). 10x nuclear lysis buffer was added to WCL and CYT fractions to equalize surfactant amounts to that of the nuclear fraction. Each resulting lysate was divided in half to facilitate either downstream protein analysis or RNA extraction.

For protein fractions, 100x Benzonase buffer (50 mM Tris-HCl, 20 mM NaCl, 2 mM MgCl_2_, 2000 U/mL Benzonase Nuclease) was added to lysates. Lysates were kept on ice for 30 minutes with periodic vortexing. The BCA assay was performed on all cellular fractions, and 10 μg of each lysate was subjected to immunoblotting as described above. Total protein was detected using the Revert 700 Total Protein Stain Kit prior to incubation with primary antibody.

For extraction of total RNA from RNA fractions, samples were subjected to the RNeasy Plus manufacturer protocol with the following modifications. 3.5x sample volume of RLT-plus buffer was added to cellular fractions. Prior to removal of genomic DNA using the gDNA Eliminator spin column, ERCC standards (diluted 1:10) were added to each lysate at a concentration of 1 μL/1 million cells (or similarly, 1 million nuclei or 1 million cell cytoplasm’s). Following genomic DNA removal, 2.5x original lysate volume of 100% ethanol was added to each flowthrough, and subsequent steps of the RNeasy Plus protocol were followed as standard. mRNA was isolated as above. Sequencing libraries were prepared with the NEBNext Ultra II Directional RNA Library Prep Kit, using manufacturer recommended modifications to generate large (∼450nt) inserts. Paired-end 2 × 150nt sequencing of libraries was performed in-house on the Illumina NextSeq2000 using the NextSeq 2000 P3 Reagent Kit.

#### STACK assay for kinetic evaluation of drug responses

The STACK assay was used to evaluate cell proliferation and cell death continuously over time using microscopy, as previously described.^13^ mKate2+ cells were seeded into 96-well black-sided optical-bottom plates, and left to adhere overnight. The following day, cells were given drugged media containing 50 nM SYTOX Green, and imaged over time time-lapsed images were collected using an IncuCyte S3 (Essen Biosciences). Images were collected with a 10x objective with acquisition in the green channel ex: 460 ± 20, em: 524 ± 20, acquisition time: 300ms; and red channel ex:585 ± 20, em: 635 ± 70, acquisition time: 400ms. Counts of dead (green) and live (red) cells were determined using the built-in IncuCyte software (Essen Biosciences) and exported for analysis using a custom MATLAB script.

For the long-term evaluation of BAX/BAK DKO cells following triptolide, accurate dead cell measurements using STACK as described above are insufficient, as identification of a dead cell from an image is no longer possible after the dead cell fully degrades away. To overcome this technical confound, a modified STACK assay was used, taking advantage of the FLICK assay (described in detail below), which does not rely on image segmentation for cell quantification. Cells were seeded and drugged as above, at a high density (15,000 cells per well). Separate plates were prepared for each timepoint, whereby cells were first imaged using an EVOS FL Auto 2 automated microscope. Images were acquired using a 10x objective (EVOS 10x objective, Cat #: AMEP4681). Sytox images were acquired using a GFP filter cube (EVOS LED Cube, GFP, Cat #: AMEP4651, ex: 470/22, em: 525/50). Mkate2+ images were acquired using a TexasRed filter cube (EVOS LED Cube TxRed, Cat #: AMEP4655, ex: 585/29, em: 628/32). Following imaging, FLICK was performed as an endpoint measurement to obtain lethal fraction values, as described below. Images were linearly adjusted using a custom MATLAB script.

#### FLICK assay for quantification of cell death

The FLICK assay was performed as described.^71,90^ Briefly, 90 μL of cells were seeded at a density of 2,000–5,000 cells per well in black-sided optical-bottom 96-well plates (Greiner Bio-One, 655090) and left to adhere overnight. The following day, cells were treated with 10 μL of media containing the indicated compound/s along with SYTOX Green, resulting in a final SYTOX concentration of 2 μM. Fluorescence was monitored at various times following drugging with a Tecan Spark (running SparkControl software version 2.2) microplate reader (ex: 503, em: 524). Gain was set to achieve a linear relationship between SYTOX signal and dead cell number. To obtain the total cell number at the time of drugging, a duplicate “T0” plate was lysed in 0.15% Triton X-100 and 2 μM SYTOX for 2–4 hours at 37C prior to taking a fluorescence reading. Total cell number for each condition at the end of the assay was similarly determined by lysing cells in 0.15% Triton X-100.

#### Pol II Switch over system

For experiments involving the RPB1 switchover system, cells were seeded as described for the respective experiment. The following day, cells we treated with or without 2 μg/mL doxycycline for 48 hours to induce the expression of FLAG-RPB1-N792D-ΔCTD. 10 μM ⍺-amanitin was added to the cells without removing the conditioned media to degrade the endogenous Rbp1.

#### Chemo-genetic profiling of triptolide

Conventional approaches used for these “chemo-genetic profiling” studies are unable to accurately identify death-regulatory genes due to the confounding effects of varied proliferation rates between cells harboring different gene knockouts.^36^ In general, specialized analytical methods are required to identify death-regulatory genes when drug-induced lethality occurs in a proliferative population, at low rates relative to proliferation, or via non-apoptotic mechanisms. However, in this case, we recognized that triptolide-induced death is: 1) exclusively apoptotic, 2) occurring rapidly (before the effects of varied proliferation can be observed in the population), and 3) activated at high rates (such that large numbers of dead cells accumulate before apoptotic corpses have decayed). Thus, we mechanically separate live cells from apoptotic corpses simply by agitating and rinsing plates to collect floating dead cells, prior to trypsinization and collection of the live cells. Both populations could then be sequenced independently.

The genome-wide CRISPR screen was performed using the Toronto KnockOut Library v3 (TKOv3), which contains 4 sgRNA per gene, along with 142 non-targeting sgRNA.^91^ U2OS-Cas9 expressing cells were selected with 5 μg/mL blasticidin for 5 days. Following selection, cells were divided into two replicates, which would be handled separately for the remainder of the assay. Cells were then transduced with the TKOv3 viral library using a ‘spinfection’ method. To maintain sufficient library coverage, more than 200 million cells were transduced per replicate, at an MOI of 0.3. To do this, cells were divided into 12-well plates at a density of 2 million cells per along with 75 μL virus and 0.8 μg/mL polybrene. Plates were centrifuged at 37 °C for 2 h at 830x*g*. Following spinfection, virus-containing media was gently removed and replaced with fresh media, and cells were returned to the incubator overnight. The following morning, cells were moved out of the 12-well plates and onto 15 cm dishes. The following day, cells were treated with 1 μg/mL puromycin for 3 days, followed by a two-day expansion without selection. Cells were then seeded onto 15cm plates and let to adhere overnight prior to drugging. At the time of drugging, a T0 sample was taken from the cell population and frozen, at a population size 650x that of the library size. The rest of the cells were treated with either 1 μM triptolide or DMSO. Cells were incubated for 32 hours in drug, resulting in approximately 50% cell death. Apoptotic corpses, which become non-adherent, were removed and saved. Plates were washed with PBS to dislodge any remaining dead cells, and PBS wash was added with the separated dead cell fraction. The adherent cells remaining on the plate were the live cell population. The live cells were trypsinized and collected. Each condition was collected such that a minimum of 650x library coverage was achieved per replicate.

Genomic DNA was extracted using the Wizard Genomic DNA Purification Kit. sgRNA sequences were PCR amplified out of the genomic DNA, and gel-extracted and purified. A second round of PCR added sequencing adaptors and multiplexing barcodes, and libraries were pooled and sequenced on a HiSeq 4000 at 500x coverage.

#### Immunofluorescence microscopy

For imaging of PTBP1 localization following drug exposure, mCherry-PTBP1 expressing cells were seeded onto round glass coverslips in 12-well plates. The following day, media was removed and replaced with media containing drug or vehicle control, along with 50 μM z-VAD-FMK. At assay endpoint, cells were washed once with PBS and fixed in 4% formaldehyde (in PBS) for 15 minutes. Cells were washed 3 times with PBS, and permeabilized in PBS-0.2% Triton-X100 for 10 minutes. Following 3 washes in PBS, cells were blocked in blocking buffer (3% BSA, 0.1% Tween20, in PBS) for 30 minutes at room temperature. Cells were then incubated at 4C overnight with anti-mCherry polyclonal Ab diluted 1:800 in blocking buffer. The following day, cells were washed 3 times with blocking buffer and incubated at room temperature for 2 hours with goat anti-rabbit IgG Alexa Fluor 594 diluted 1:1000 in blocking buffer. Cells were washed 3 times with blocking buffer, once with PBS, then incubated at room temperature for 20 minutes with CoraLite Plus 488-Phalloidin (1:200 in PBS). Following 2 washes with PBS, cells were stained with DAPI and mounted on glass slides with Prolong Diamond.

For imaging of BCL2L12 localization following Rpb1 degradation, BCL2L12-Flag-HA expressing cells were seeded onto round glass coverslips in 12-well plates. The following day, media was removed and replaced with media containing drug or vehicle control, along with 50 μM z-VAD-FMK. At assay endpoint cells were fixed and permeabilized as above. Cells were then incubated at 4C overnight with anti-HA Tag mAb and anti-Tom20 mAb diluted 1:500 and 1:200 in blocking buffer, respectively. The following day, cells were washed 3 times with blocking buffer and incubated at room temperature for 2 hours with goat anti-mouse IgG1 Alexa Fluor 488 and goat anti-rabbit IgG Alexa Fluor 594 diluted 1:2000 and 1:1000 in blocking buffer, respectively. Cells were washed 3 times with blocking buffer, stained with DAPI, and mounted on glass slides with Prolong Diamond.

IF slides were imaged using an EVOS FL Auto 2 automated microscope. Images were acquired using a 40x objective (EVOS 40x objective, Cat #: AMEP4699). Images were acquired using a GFP filter cube (EVOS LED Cube GFP, Cat #: AMEP4651, ex: 470/22, em: 525/50), a TexasRed filter cube (EVOS LED Cube TxRed, Cat #: AMEP4655, ex: 585/29, em: 628/32), and a DAPI filter cube (EVOS LED Cube DAPI, Cat #: AMEP4650, ex: 357/44, em: 447/60).

#### iBH3 profiling assay

Cytochrome C release in cells following exposure to peptides was performed using the iBH3 profiling assay.^55^ Human BIM-BH3 (N term – MRPEIWIAQELRRIGDEFNA – C term), and human W-BCL2L12-BH3-like (N term – WEAILRRLVALLEEEAEVINQ – C term) peptides were synthesized through Genscript (purity >= 95%; N-term acetylation; C-term amidation). A tryptophan (W) residue was added to the N-terminus of the BCL2L12-BH3-like sequence (as shown above) to facilitate UV absorbance measurements. Peptides were dissolved in DMSO, and quantified by UV absorption at 280 nm. U2OS, U2OS-BAX/BAK-DKO, U2OS-PTBP1-KO, and U2OS-BCL2L12-KO cells were collected through trypsinization and pelleting, and resuspended at 2 million cells per mL in MEB2 buffer (150mM Mannitol, 10mM HEPES-KOH pH 7.5, 150mM KCl, 1mM EGTA, 1mM EDTA, 0.1% BSA, 5mM Succinate). 50 μL cells were then added to 50 μL of treatment, diluted in MEB2 buffer containing 0.002% digitonin. 15 μM alamethicin was used as a positive control, and 0.1% DMSO was used as a negative control. Treatment was terminated after 1 hour by addition of 33 μL of 4% formaldehyde (in PBS) for 10 minutes. Fixation was terminated by addition of 33 μL of N2 buffer (1.7 M Tris base, 1.25 M Glycine, pH 9.1) for 5 minutes. 20μL of Alexa Fluor 488 anti-cytochrome c (clone 6H2.B4) diluted in 10X CytoC stain buffer (10% BSA and 2% Tween20 in PBS) was then added to samples, reaching a final antibody dilution of 1:400. Samples were incubated with antibody overnight at 4C, washed once with PBS-0.1% Tween, filtered, and run on a Miltenyi MACSQuant VYB cytometer with laser and filter settings appropriate for reading Alexa-488. Data were analyzed using FlowJo software.

#### Flow Cytometry

For the quantification of cytochrome C release following treatment with triptolide, U2OS, U2OS-PTBP1-KO, and U2OS-BCL2L12-KO cells were plated in 6-well plates such that there would be 300,000 cells at the time of collection. After adhering overnight, cells were treated with 1 μM tripolide or vehicle control, along with 50 μM z-VAD-FMK, for 24 hours. Cells were then collected through trypsinization and pelleting. Cells were resuspended in MEB2 buffer containing 0.001% digitonin and incubated for 10 minutes. Cells were then fixed, stained, and analyzed as described for the iBH3 protocol above.

For the quantification of exogenous BCL2L12-Flag-HA protein expression in U2OS-BCL2L12-KO cells, 300,000 cells expressing either BCL2L12-Flag-HA or BCL2L12-E220A-Flag-HA were trypsinized and pelleted. Cells were washed with PBS, fixed in 4% formaldehyde (in PBS) for 15 minutes, washed once with PBS, and permeabilized with 100% cold methanol. Cells were stored overnight at −20C. The following day, cells were washed twice with PBS-0.1% Tween, then incubated at 4C overnight with anti-HA Tag mAb (2-2.2.14) diluted 1:500 in 1% BSA (in PBS). The following day, cells were washed once with PBS-0.1% Tween, then incubated for 2 hours with goat anti-mouse IgG Alexa Fluor 488 (diluted 1:500 in 1% BSA). Cells were washed two more times with PBS-0.1% Tween, resuspended in PBS-0.1% Tween, filtered, and run on a Miltenyi MACSQuant VYB cytometer with laser and filter settings appropriate for reading Alexa-488. Data were analyzed using FlowJo software.

#### Evaluation of drug PDS score

FLICK was used to determine lethal fraction kinetics in U2OS, PTBP1-KO, BCL2L12-KO, and BAX/BAK DKO cells across a panel of 46 drugs, across 7 half-log dilutions, and spanning the following 16 classes. Pol II degraders (Triptolide, 1 – 0.001 μM; ⍺-amanitin, 10 – 0.01 μM). RNA synthesis inhibitors (Actinomycin-D, 1 – 0.001 μM; Ethynylcytidine, 10 – 0.01 μM; Cordycepin, 100 – 0.1 μM). Transcriptional CDK inhibitors (Flavopiridol, 10 – 0.01 μM; DRB, 100 – 0.1 μM; THZ1, 10 – 0.01 μM; THZ531, 10 – 0.01 μM; YKL-5-124, 31.6 – 0.0316 μM). Cell cycle CDK inhibitors (Abemaciclib, 10 – 0.01 μM; Palbociclib, 10 – 0.01 μM; Ribociclib, 10 – 0.01 μM; PF3600, 31.6 – 0.0316 μM; PF4091, 31.6 – 0.0316 μM). DNA crosslinking and nucleotide damaging (4-NQO, 31.6 – 0.0316 μM; Cisplatin, 100 – 0.1 μM; Carboplatin, 316 – 0.316 μM). Topoisomerase I inhibitors (Camptothecin, 10 – 0.01 μM; Topotecan, 31.6 – 0.0316 μM). Topoisomerase II inhibitors (Idarubicin, 1 – 0.001 μM; Etoposide, 31.6 – 0.0316 μM; Teniposide, 31.6 – 0.0316 μM). PARP inhibitors (Niraparib, 100 – 0.1 μM; Rucaparib, 100 – 0.1 μM). Translational inhibitors (Cycloheximide, 100 – 0.1 μM; Homoharringtonine, 10 – 0.01 μM; Anisomycin 316 – 0.316 μM). Proteasome inhibitors (Bortezomib, 1 – 0.001 μM; MG132, 10 – 0.01 μM). ER- and proteotoxic-stress inducers (Brefeldin A, 31.6 – 0.0316 μM; Thapsigargin, 31.6 – 0.0316 μM; Tuni-camycin, 10 – 0.01 μM). BH3 mimetics (ABT-199, 100 – 0.1 μM; ABT-263, 100 – 0.1 μM; ABT-737, 31.6 – 0.0316 μM). HDAC inhibitors (Panobinostat, 1 – 0.001 μM; Vorinostat, 100 – 0.1 μM). mTOR inhibitors (Torin2, 31.6 – 0.0316 μM; Rapamycin, 10 – 0.01 μM). MEK/PI3K/AKT inhibitors (Mirdametinib, 31.6 – 0.0316 μM; Buparlisib, 31 – 0.0316 μM; Capivasertib, 31.6 – 0.0316 μM). Others (SGI-1027, 31.6 – 0.0316 μM; JQ1, 100 – 0.1 μM; Staurosporine, 10 – 0.01 μM).

Lethal fraction kinetics were fit to a lag exponential death (LED) equation. Non-lethal conditions were defined as those having a maximum observed lethal fraction less than 0.16, approximately double the death observed in untreated cells. Cells were further classified as apoptotic or non-apoptotic by thresholding a 50% reduction in maximum lethal fraction in the BAX/BAK DKO background compared to U2OS cells. To determine the “Pol II Degrader Similarity” (PDS) score for a particular drug-dose pair, the area under the LED fit (AUC) was determined, and the baseline cell death was removed by subtracting out the AUC of untreated cells. The difference in drug response between KO and U2OS cells was defined as 1 minus the AUC ratio of KO over U2OS. This value, determined for PTBP1-KO and BCL2L12-KO separately, was summed. Finally, scores were divided by that of 1 μM triptolide, such that a score of one denotes a functional genetic signature equal to that of triptolide.

#### Evaluation of genetic dependencies across cells

In order to evaluate the dependence of cell death on BAX/BAK1, PTBP1, and BCL2L12 across different cell lines, the in4mer system was used to generate genetic knockouts (described above). 90 μL of selected cells were seeded at a density of 2,000–5,000 cells per well in black-sided optical-bottom 96-well plates (Greiner Bio-One, 655090) and left to adhere overnight. The following day, cells were treated with 10 μL of media containing the indicated compound/s along with 5 μM SYTOX Green, and subjected to the FLICK assay (described above). Assay length was varied based on the cell death kinetics of each cell line, such that greater than 50% cell death was observed by assay endpoint.

### QUANTIFICATION AND STATISTICAL ANALYSIS

#### RNA sequencing analysis

Quality control for the RNAseq datasets were performed using FastQC. Transcript abundance was estimated using Kallisto with parameters –bootstrap-samples 30 –single-overhang –rf-stranded. The Ensembl GRCh38 cDNA transcriptome build, appended with ERCC control transcript sequences, was used to build a Kallisto index. Gene-level counts were generated in R using the txim-port package. Counts were normalized to ERCC spike-ins using DESeq2, implemented with the function “estimateSizeFactors” with the option “controlGenes” set to the identities of the 92 ERCC transcripts. Counts were filtered for protein coding genes in R using AnnotationHub. Differential gene expression was performed with DESeq2, and fold change shrinkage was performed using the function lfcShrink with the adaptive shrinkage estimator “ashr.”^92^ For comparative gene-level analysis between U2OS cells and PTBP1-KO whole cell extracts, the cytoplasmic and nuclear RNAseq FASTQ files corresponding to the same biological samples were pooled. For all analysis related to the quantification of mRNA decay following transcriptional inhibition, genes were filtered to include those with reliably detectable expression in untreated cells (cutoff determined from ERCC spike-ins, mean normalized count of 10).

To facilitate detection of splice-level events, cytoplasmic and nuclear RNAseq FASTQ files corresponding to the same biological samples (above) were pooled. Regulated splicing events were detected and analyzed with VAST-TOOLS. Briefly, reads were aligned to the human genome (hg38) using the align function. Differential splicing events were identified using the diff function (with parameters -S 2). Events were filtered for adequate coverage using the tidy function (with parameters -min_N 4, –noVLOW, –min_SD 5, and –p_IR). Splicing events were considered significantly different if the VAST-TOOLS reported expected absolute ΔPSI value was greater than 0.1 and if the minimum value (95% confidence) of the absolute ΔPSI value was greater than 0.

#### Analysis of growth and death data from the FLICK assay

From measurements made from FLICK (dead cells at each timepoint, along with total cell numbers at the beginning and end of the assay), live cell numbers over time can be inferred and all calculations to determine LF, FV, RV and GR can be made^65^:

$$LF=\frac{Dead}{Live+Dead},FV=\frac{Live}{Live+Dead},RV=\frac{{Live}_{Trt}}{{Live}_{Unt}},GR=2^{\left(\frac{{log}_{2}\left(\frac{{Live}_{Trt}}{{Live}_{T0}}\right)}{{log}_{2}\left(\frac{{Live}_{Unt}}{{Live}_{T0}}\right)}\right)}-1$$

All analyses were performed using custom MATLAB scripts. Dose-response curves were fit using a 4-parameter sigmoidal equation. Lethal fraction (LF) kinetics were fit using the lag-exponential death (LED) equation, where LF_0_ is the initial LF in the absence of drug, LF_p_ is the asymptotic LF in the exponential fit, D_R_ is the initial death rate, D_O_ is the death onset time, and *t* is time in hours^13^:

$$LF\left(t\right)={LF}_{0}+({LF}_{P}-{LF}_{0})(1-e^{-D_{R}(t-D_{O})})$$

All analyses were performed using custom MATLAB scripts. All data were fit with the “fit” function in MATLAB using nonlinear least-squares.

#### Drug GRADE-based analysis of drug responses

True proliferation rates and death rates were calculated using the GRADE method using STACK data, as previously described.^65^ Conceptually, the GRADE method infers the true, underlying cell proliferation rates and death rates of cell population by cross-referencing experimentally measured death rates (FV or LF) and net population growth rates (GR) with computational simulations. To begin, we simulated all pairwise combinations of 500 proliferation rates and 500 death rates using the following equations where *C*_*0*_ = initial cell number, *t* = assay length, *τ* = proliferation rate, and *D*_*R*_ = death rate.


$$Live\;Cell\;Number=\left(C_{0}*2^{\left(\frac{t}{\tau }\right)}\right)-(C_{0}*2^{\left(\frac{t}{\tau }\right)}*D_{R})$$

$$Dead\;Cell\;Number=C_{0}*2^{\left(\frac{t}{\tau }\right)}*D_{R}$$

FV and GR values (defined above) were then calculated for each simulated proliferation rate and death rate pair. Next, for a given condition, experimentally determined FV and GR values were calculated (as above). The simulated proliferation rate and death rate that yield the empirically determined FV and GR values was then found.

#### Quantification of Pol II half-life

Half-lives for Pol IIA and Pol IIO were determined using temporally resolved quantitating immunoblotting using an antibody recognizing the N terminal domain of Rpb1 (anti-Rpb1-NTD, clone D8L4Y), as described above. Pol II protein primarily exists in two forms. Pol IIO is hyper-phosphorylated along the carboxy-terminal domain (CTD) of the protein and is the actively elongating pool of Pol II. The hypo-phosphorylated form, called Pol IIA, represents all other forms of Pol II that are not actively elongating RNA, including preinitiation complex (PIC) bound, promoter paused, early pause released, and free Pol II. Pol IIO and Pol llA can be distinguished by a band shift on a gel. Immunoblots were quantified in ImageStudio, normalized to β-actin, then normalized to the untreated sample (time 0) on each gel respectively, generating an abundance value relative to steady state. Data were fit to a two-term exponential decay function in MATLAB, relative to the highest Rpb1 signal observed over the time course. Half-lives determined from the fits, as the time at which half-maximal signal was reached. To correlate Pol II half-lives with cell death timing, the LF50 time was similarly found from lag-exponential death (LED) fits to STACK data. LF50 was defined as the time at which half of the maximum observed LF (measured or inferred after 72 hours) was reached. For identifying the timing of loss of nascent transcription, EU measurements (as described above), were fit to a 4-parameter hill function. The EU t_1/2_ values were determined from the fits as the time at which 50% of cells were EU negative.

#### Chemo-genetic profiling analysis

The FASTX-Toolkit was used to trim reads to get just CRISPR guide sequences (parameters: -f 23 -l 42). Trimmed reads were mapped to the TKOv3 library with Bowtie allowing for 2 mismatches (parameters: -v 2 -m 1). Samples were normalized for sequencing depth using median of ratios method implemented in DESeq2. Guides with low counts (approximately the bottom 2% of guides) were removed from the library. sgRNA-level log_2_ fold-changes were determined using DESeq2 and were then z-scored to the distribution of nontargeting sgRNA. The median sgRNA-level z-score was determined for each gene to collapse scores to the gene level. An empiric *P* value was determined for each gene by bootstrapping from the sgRNA-level fold change scores of all guides, and was FDR corrected using the Benjamini–Hochberg procedure. An FDR cutoff of 0.1 was used to determine genes that significantly alter the cell death rate following triptolide. Gene set enrichment using an FDR-corrected one-tailed Fishers Exact test was performed to test if any apoptotic-related pathways from the Molecular Signatures Database (MSigDB) were overrepresented among genes whose knockout significantly reduced triptolide-induced death. To avoid confounded results, core apoptotic regulators (defined as those in the Biocarta Mitochondrial Pathway gene set) were removed from all other gene sets analyzed. To identify if fast or slow decaying mRNAs are preferentially enriched at the extreme ends of our chemo-genetic profiling data, GSEA was run with a pre-ranked list of the relative death rate scores. Gene sets were defined at the top 1% fastest or slowest decaying mRNAs (133 genes each), based on our RNAseq data.

#### Drug PDS score definition

Lethal fraction kinetics were fit to a lag exponential death (LED) equation. Non-lethal conditions were defined as those having a maximum observed lethal fraction less than 0.16, approximately double the death observed in untreated cells. To determine the “Pol II Degrader Similarity” (PDS) score for a particular drug-dose pair, the area under the LED fit (AUC) was determined, and the baseline cell death was removed by subtracting out the AUC of untreated cells. The difference in drug response between KO and U2OS cells was defined as 1 minus the AUC ratio of KO over U2OS. This value, determined for PTBP1-KO and BCL2L12-KO separately, was summed. Finally, scores were divided by that of 1 μM triptolide, such that a score of one denotes a functional genetic signature equal to that of triptolide.

Evaluation of genetic dependance across cell lines using the in4mer system of cell death on BAX/BAK1, on PTBP1, on BCL2L12, as well as PDS scores across cell lines, followed an identical approach. Scores were further normalized to that of triptolide in U2OS cells as a validated control.

#### Probabilistic drug classifier

Drugs were classified as “triptolide-like” based on their dependency on PTBP1, BCL2L12, BAX, and BAK1 for causing cell death. To perform this functional genetic classification, we adapted an established approach used previously.^59,60^ Briefly, for each drug-dose pair, AUC ratios between each KO and U2OS were determined. This places each drug-dose pair in a 3-dimensional functional genetic space, defined by the relative AUCs observed in PTBP1-KO vs. WT, BCL2L12-KO vs. WT, and BAX/BAK DKO vs. WT. The “neighborhood” of transcriptional inhibitors was calculated as the mean of pairwise Euclidean distances between validated Pol II degraders (triptolide and ⍺-amanitin) in this 3-dimensional space. The linkage ratio describes the similarity of a query compound to triptolide and ⍺-amanitin and defines how much the neighborhood expands or shrinks after the query compound is included in it. A set of negative controls, defined as a set of drugs with known mechanism of action distinct from transcriptional inhibition, is then forced to be included in the transcriptional inhibitor neighborhood, iteratively, causing the neighborhood to expand or shrink. BH3 mimetics (ABT-199, ABT-263, ABT-737), Topoisomerase inhibitors (Etoposide, Camptothecin, Idarubicin, Topotecan, and Teniposide), and ER-stress inducers (Thapsigargin, Tunicamycin, and Brefeldin A) were used to generate the false expansion. This process of “false expansion” generates an empirically defined null distribution of linkage ratios. A linkage ratio for each drug-dose pair is then determined and compared to the null distribution to derive a *P* value. *P* values are then FDR corrected using the Benjamini–Hochberg procedure.

#### IF image analysis

Images were analyzed in Fiji. DAPI staining was used to mask nuclei, and a cytoplasmic area was drawn for each analyzed cell using actin staining. Images were quantified in a double-blinded fashion. Raw intensities within each region of interest were area normalized, and cytoplasmic/nuclear ratios for each cell were calculated. For quantifying nuclear versus mitochondrial intensity, Tom20 staining was used to mask mitochondria.

#### Data analysis and statistics

Unless otherwise noted, data analysis and statistics was performed in MATLAB (version R2024b) using built-in functions. Bar plots were generated in GraphPad Prism 10. ImageStudio 3.1.4 was used to analyze western blots. Flow cytometry analysis was performed using FlowJo version 10.8.1 software. Details of statistical tests—including type of test used, exact values of *n*, definition of center, dispersion and precision measurements, and definitions of significance—are indicated in the respective figure legends and methods section.

## Supplementary Material

MMC4

MMC2

MMC1

MMC3

MMC7

MMC6

MMC5

8

Supplemental information can be found online at https://doi.org/10.1016/j.cell.2025.07.034.

## Figures and Tables

**Table:** KEY RESOURCES TABLE

REAGENT or RESOURCE	SOURCE	IDENTIFIER
Antibodies		
Anti-Rpb1-NTD rabbit mAb (clone D8L4Y)	Cell Signaling Technology	Cat# 14958; RRID:AB_2687876
Anti-DYKDDDDK Tag (FLAG) rabbit mAb	Cell Signaling Technology	Cat# 14793S; RRID:AB_2572291
Anti-β-actin mouse mAb	Sigma-Aldrich	Cat# A2228; RRID:AB_476697
Anti-Ptbp1 rabbit mAb	Cell Signaling Technology	Cat# 57246S; RRID:AB_2799528
Anti-Histone-H3 rabbit mAb	Cell Signaling Technology	Cat# 4499S; RRID:AB_10544537
Anti-⍺-tubulin mouse mAb	Sigma-Aldrich	Cat# T6199; RRID: AB_477583
Anti-pH2A.X (Ser139) rabbit mAb	Cell Signaling Technology	Cat# 9718; RRID:AB_2118009
Anti-Mcl-1 rabbit mAb	Cell Signaling Technology	Cat# 5453; RRID:AB_10694494
Anti-Apaf-1 rabbit mAb	Cell Signaling Technology	Cat# 8723S; RRID:AB_10829610
Anti-β-actin rabbit polyclonal Ab	Sigma-Aldrich	Cat# A2066; RRID:AB_476693
IRDye 680RD goat anti-mouse IgG	LI-COR	Cat# 926–68070; RRID:AB_10956588
IRDye 800CW goat anti-rabbit IgG	LI-COR	Cat# 926–32211; RRID:AB_621843
Anti-Bax rabbit mAb (clone D2E11)	Cell Signaling Technology	Cat# 5023; RRID:AB_10557411
Anti-Bax mouse mAb (clone 6A7)	BD Biosciences	Cat# 556467; RRID:AB_396430
Mouse IgG1, κ isotype control mAb	BioLegend	Cat# 400101; RRID: AA_2891079
Anti-Rpb1 mouse mAb (clone 8WG16)	BioLegend	Cat# 664906; RRID:AB_2565554
Anti-phospho-Rpb1 CTD (Ser2) rabbit mAb	Cell Signaling Technology	Cat# 13499; RRID:AB_2798238
Anti-phospho-Rpb1 CTD (Ser5) rabbit mAb	Cell Signaling Technology	Cat# 13523; RRID:AB_2798246
Anti-phospho-Rpb1 CTD (Ser7) rabbit mAb	Cell Signaling Technology	Cat# 13780; RRID:AB_2687900
Anti-mCherry rabbit polyclonal Ab	Proteintech	Cat# 26765–1-AP; RRID: AB_2876881
Anti-Tom20 rabbit mAb	Cell Signaling Technology	Cat# 42406; RRID:AB_2687663
Mouse IgG2a, κ isotype control mAb	BioLegend	Cat# 400201; RRID: AB_2927399
Anti-HA Tag mouse mAb	Thermo Fisher Scientific	Cat# 26183; RRID:AB_10978021
Alexa Fluor 488 anti-cytochrome c mouse mAb (clone 6H2.B4)	BioLegend	Cat# 612308; RRID:AB_2565240
Anti-c-Myc rabbit mAb	Cell Signaling Technology	Cat# 5605; RRID:AB_1903938
goat anti-rabbit IgG Alexa Fluor 594	Thermo Fisher Scientific	Cat# A-11012; RRID:AB_2534079
goat anti-mouse IgG1 Alexa Fluor 488	Thermo Fisher Scientific	Cat# A-21121; RRID:AB_2535764
goat anti-mouse IgG Alexa Fluor 488	Thermo Fisher Scientific	Cat# A-11001; RRID:AB_2534069
Bacterial and virus strains		
NucLight Red Lentivirus	Sartorius	Cat# 4627
Chemicals, peptides, and recombinant proteins		
phosSTOP phosphatase inhibitor tablets	Sigma-Aldrich	Cat# 4906837001
cOmplete, EDTA-free Protease Inhibitor Cocktail	Sigma-Aldrich	Cat# 11873580001
Intercept Blocking Buffer	LI-COR	Cat# 927–70003
5-ethynyl uridine	Sigma-Aldrich	Cat# 909475
Formaldehyde solution (36.5–38%)	Sigma-Aldrich	Cat# F8775
polyadenylated ERCC standards	Thermo Fisher Scientific	Cat# 4456740
SYTOX Green	Thermo Fisher Scientific	Cat# S7020
Triptolide	Sigma-Aldrich	Cat# T3652
⍺-amanitin	Sigma-Aldrich	Cat# A2263
ABT-737	ApexBio	Cat# A8193
RSL3	ApexBio	Cat# B6095
Elesclomol	Sigma-Aldrich	Cat# SML2651
MNNG	Biosynth Carbosynth	Cat# FM11256
TNF-⍺	ApexBio	Cat# P1001
SM-164	ApexBio	Cat# A8815
IAA	Sigma-Aldrich	Cat# I5148
z-VAD-FMK	ApexBio	Cat# A1902
VX765	ApexBio	Cat# A8238
TTM	Sigma-Aldrich	Cat# 323446
Rucaparib	ApexBio	Cat# A4156
Necrostatin-1	SelleckChemicals	Cat# S8037
Ferrostatin-1	SelleckChemicals	Cat# S7243
Doxycycline Hyclate	Sigma-Aldrich	Cat# D5207
Actinomycin-D	SelleckChemicals	Cat# S8964
Ethynylcytidine	MedChemExpress	Cat# HY-16200
Cordycepin	Sigma-Aldrich	Cat# C3394
Flavopiridol	SelleckChemicals	Cat# S1230
DRB	Sigma-Aldrich	Cat# D1916
THZ1	SelleckChemicals	Cat# S7549
THZ531	MedChemExpress	Cat# HY-103618
YKL-5–124	MedChemExpress	Cat# HY-101257B
Abemaciclib	MedChemExpress	Cat# HY-16297A
Palbociclib	ApexBio	Cat# A8316
Ribociclib	MedChemExpress	Cat# HY-15777
PF3600	SelleckChemicals	Cat# S8816
PF4091	ChemieTek	Cat# CT-PF0710
4-NQO	SelleckChemicals	Cat# E0155
Cisplatin	SelleckChemicals	Cat# S1166
Carboplatin	Sigma-Aldrich	Cat# C2538
Camptothecin	SelleckChemicals	Cat# S1288
Topotecan HCl	SelleckChemicals	Cat# B2296
Idarubicin HCl	SelleckChemicals	Cat# S1228
Etoposide	SelleckChemicals	Cat# S1225
Teniposide	SelleckChemicals	Cat# S1787
Niraparib	SelleckChemicals	Cat# S2741
Rucaparib phosphate	SelleckChemicals	Cat# S1098
Cycloheximide	Sigma-Aldrich	Cat# C1988
Homoharringtonine	Sigma-Aldrich	Cat# SML1091
Anisomycin	Sigma-Aldrich	Cat# A9789
Bortezomib	ApexBio	Cat# A2614
MG-132	SelleckChemicals	Cat# S2619
Brefeldin A	Sigma-Aldrich	Cat# B7651
Thapsigargin	Sigma-Aldrich	Cat# T9033
Tunicamycin	Sigma-Aldrich	Cat# T7765
ABT-199	ApexBio	Cat# A8194
ABT-263	ApexBio	Cat# A3007
Panobinostat	ApexBio	Cat# A8178
Vorinostat	ApexBio	Cat# A4084
Torin 2	ApexBio	Cat# B1640
Rapamycin	ApexBio	Cat# A8167
Mirdametinib	MedChemExpress	Cat# HY-10254
Buparlisib	MedChemExpress	Cat# HY-70063
Capivasertib	MedChemExpress	Cat# HY-15431
SGI-1027	ApexBio	Cat# B1622
JQ1	SelleckChemicals	Cat# S7110
Staurosporine	SelleckChemicals	Cat# S1421
H2O2	Fisher Scientific	Cat# BP2633500
LPS	Sigma-Aldrich	Cat# L4391
Nigericin	ApexBio	Cat# B7644
copper(II) chloride	Thermo Fisher Scientific	Cat# 405845000
S63845	SelleckChemicals	Cat# S8383
G418	Sigma-Aldrich	Cat# G8168
CoraLite Plus 488-Phalloidin	Proteintech	Cat# PF00001
SUPERase-In RNase inhibitor	Thermo Fisher Scientific	Cat# AM2694
Benzonase Nuclease	Sigma Aldrich	Cat# E1014
Alamethicin	MedChemExpress	Cat# HY-N6708
Dimethyl Sulfoxide (DMSO)	Fisher Scientific	Cat# MT-25950CQC
Critical commercial assays		
Proteome Profiler Apoptosis Arrays	R&D Systems	Cat# ARY009
Gibson Assembly Master Mix	New England Biolabs	Cat# E2611
NEBridge Golden Gate Assembly Kit (BsmBI-v2)	New England Biolabs	Cat# E1602
Gateway LR Clonase II Enzyme mix	Thermo Fisher Scientific	Cat# 11791020
Pierce BCA Protein Assay Kit	Thermo Fisher Scientific	Cat# 23227
FuGENE HD Transfection Reagent	Promega	Cat# E2311
QIAquick PCR Purification Kit	Qiagen	Cat# 28104
Rneasy Plus Mini Kit	Qiagen	Cat# 74134
Click-iT RNA Alexa Fluor 488 Imaging Kit	Thermo Fisher Scientific	Cat# C10329
NEBNext Poly(A) mRNA Magnetic Isolation Module	New England Biolabs	Cat# E7490
NEBNext Ultra II Directional RNA Library Prep Kit	New England Biolabs	Cat# E7765
NEBNext Multiplex Oligos for Illumina	New England Biolabs	Cat# E7600
NextSeq 1000/2000 P^2^ XLEAP-SBS Reagent Kit	Illumina	Cat# 20100987
NextSeq 2000 P3 Reagent Kit	Illumina	Cat# 20040561
CellTiter Glo	Promega	Cat# G7571
QIAquick Gel Extraction Kit	Qiagen	Cat# 28704
Wizard Genomic DNA Purification Kit	Fisher Scientific	Cat# PR-A1125
Q5 Site-Directed Mutagenesis Kit	New England Biolabs	Cat# E0554S
Pierce Protein G Magnetic Beads	Thermo Fisher Scientific	Cat# 88847
Chromotek RFP-Trap magnetic agarose	Proteintech	Cat# rtma
Quick Western Kit	LI-COR	Cat# 926–69100
Revert 700 Total Protein Stain Kit	LI-COR	Cat# 926–11010
Polybrene	Millipore	Cat# TR1003G
Prolong Diamond antifade mountant	Thermo Fisher Scientific	Cat# P36965
Deposited data		
RNAseq: triptolide exposure time course in U2OS and BAX/BAK DKO cells	This study	Gene Expression Omnibus (GEO) accession number: GSE283148
RNAseq: triptolide exposure in NUP93 or EXOSC5 KO cells	This study	Gene Expression Omnibus (GEO) accession number: GSE283149
RNAseq: nuclear and cytoplasmic fractionation in U2OS or PTBP1 KO cells	This study	Gene Expression Omnibus (GEO) accession number: GSE283150
RNAseq: triptolide exposure following a-amanitin in Pol II switchover system	This study	Gene Expression Omnibus (GEO) accession number: GSE283151
Chemo-genetic profiling data	This study	Gene Expression Omnibus (GEO) accession number: GSE283147
Experimental models: Cell lines		
U-2-OS (U2OS)	ATCC	Cat# HTB-96
HT-29	ATCC	Cat# HTB-38
H1650	ATCC	Cat# CRL-5883
WI-38	ATCC	Cat# CCL-75
Hs578T	ATCC	Cat# HTB-126
MDA-MB-453	ATCC	Cat# HTB-131
MCF7	ATCC	Cat# HTB-22
MDA-MB-157	ATCC	Cat# HTB-24
HeLa	ATCC	Cat# CCL-2
MCF10A	ATCC	Cat# CRL-10317
A375	Michael Green lab, UMass Chan Medical School	N/A
HAP1	Job Dekker lab, UMass Chan Medical School	N/A
HAP1-RPB1-AID	Valton et al.^19^	N/A
RPE-1	Gregory Pazour lab, UMass Chan Medical School	N/A
PC9	Justin Pritchard lab, Penn State	N/A
Lenti-X 293T	Takara	Cat# 632180
U2OS-BAX/BAK-DKO	Richards et al.^71^	N/A
U2OS-mKate2+	Richards et al.^71^	N/A
U2OS-BAX/BAK-DKO-mKate2+	This paper	N/A
U2OS-PTBP1-KO	This paper	N/A
U2OS-BCL2L12-KO	This paper	N/A
U2OS-Rpb1-N792D-ΔCTD Switchover	This paper	N/A
U2OS-mCherry-PTBP1	This paper	N/A
U2OS-BCL2L12-Flag-HA	This paper	N/A
U2OS-Cas9	Honeywell et al.^36^	N/A
U2OS-TP53-KO	Honeywell et al.^36^	N/A
Oligonucleotides		
pLCKO2::TKOv3 library	Hart et al.,^35^ Addgene	Cat# 125517
Primers for PTBP1 knockout validation: Forward primer (same as sequencing primer): 5’-gatgctgaaggggaa aaaccaggtac-3’; Reverse primer: 5’-tcttcaacactgtgccgaacttggag-3’	This paper, Integrated DNA Technologies	N/A
Primers for BCL2L12 knockout validation: Forward primer (same as sequencing primer): 5’-cagctactccagacttctatgctttg-3’; Reverse primer: 5’-acggtacagtactaccctaaccatac-3’	This paper, Integrated DNA Technologies	N/A
sgRNA sequences (Table S1)	This paper, Integrated DNA Technologies	N/A
gBlocks for in4mer constructs (Table S1)	This paper, Integrated DNA Technologies	N/A
PCR primers for cloning Rpb1 Switchover system: Forward primer: 5’ –CAATTCCACAACACTTTTGTC TTATACTTGGATCCATGGACTACAAGGACGACGATGACA −3’; Reverse primer:	This paper, Integrated DNA Technologies	N/A
5’ –TAGGGGGGGGGGAGGGAGAGGGGC CGGCCGGGGCTCAGCTGGGAGACATGGCACCAC–3’		
PCR#1 pLCKO2-TKOv3 library forward: 5’- GAGGGCCTATTTCCCATGATTC-3’	Hart et al.,^35^ Integrated DNA Technologies	N/A
PCR#1 pLCKO2-TKOv3 library reverse: 5’- CAAACCCAGGGCTGCCTTGGAA-3’	Hart et al.,^35^ Integrated DNA Technologies	N/A
Recombinant DNA		
Plasmid: pX330-PTBP1-Puro	This paper	N/A
Plasmid: pX330-BCL2L12-Puro	This paper	N/A
Plasmid: pX330-NUP93-Puro	This paper	N/A
Plasmid: pX330-EXOSC5-Puro	This paper	N/A
Plasmid: pX330-MCL1-Puro	This paper	N/A
Plasmid: pX330-MYC-Puro	This paper	N/A
Plasmid: pX330-WEE1-Puro	This paper	N/A
Plasmid: pX330-TICRR-Puro	This paper	N/A
Plasmid: pX330-JUN-Puro	This paper	N/A
Plasmid: pX330-FBXO5-Puro	This paper	N/A
Plasmid: pX330-DTL-Puro	This paper	N/A
Plasmid: pX330-DBR1-Puro	This paper	N/A
Plasmid: pX330-BCL2A1-Puro	This paper	N/A
Plasmid: pX330-NONT-Puro	This paper	N/A
Plasmid: pRDA_550-PTBP1	This paper	N/A
Plasmid: pRDA_550-BCL2L12	This paper	N/A
Plasmid: pRDA_550-BAX/BAK1	This paper	N/A
Plasmid: pRDA_550-Intergenic	This paper	N/A
Plasmid: pLVX-Tre3G-FLAG-RPB1-N792D-ΔCTD	This paper	N/A
Plasmid: pLVX-Tet3G	Clontech	Cat# 631358
Plasmid: pHAGE-mCherry-PTBP1-IRES-Puro	This paper	N/A
Plasmid: pHAGE-BCL2L12-FLAG-HA-IRES-Puro	This paper	N/A
Plasmid: pHAGE-BCL2L12-E220A-FLAG-HA-IRES-Puro	This paper	N/A
Software and algorithms		
TIDE (version 3.3.0)	Brinkman et al.^72^	https://tide.nki.nl/
ImageStudio software (version 3.1.4)	LI-COR	https://www.licorbio.com/image-studio
FlowJo software (version 10.8.1)	FlowJo	https://www.flowjo.com/
FastQC (version 0.12.1)	Babraham Bioinformatics	https://www.bioinformatics.babraham.ac.uk/projects/fastqc/
Kallisto (version 0.46.1)	Bray et al.^73^	https://pachterlab.github.io/kallisto/
Tximport (version 1.16.1)	Soneson et al.^74^	https://bioconductor.org/packages/release/bioc/html/tximport.html
DESeq2 (version 1.28.1)	Love et al.^75^	https://bioconductor.org/packages/release/bioc/html/DESeq2.html
AnnotationHub (version 2.20.2)	Morgan and Shepherd^76^	https://bioconductor.org/packages/release/bioc/html/AnnotationHub.html
VAST-TOOLS	Irimia et al.^77^ and Tapial et al.^78^	https://github.com/vastgroup/vast-tools
FASTX-Toolkit (version 0.0.14)	N/A	https://github.com/agordon/fastx_toolkit
Bowtie (version 1.3.0)	Langmead et al.^79^	https://bowtie-bio.sourceforge.net/index.shtml
GSEA software (version 4.2.2)	Subramanian et al.^80^	https://www.gsea-msigdb.org/gsea/index.jsp
Fiji software (version 2.9.0)	Schindelin et al.^81^	https://imagej.net/software/fiji/
MATLAB software (version R2024b)	MathWorks	https://www.mathworks.com/products/matlab.html
Prism 10 software	GraphPad	https://www.graphpad.com/
IncuCyte Software (2023a)	Essen Biosciences	https://www.sartorius.com/en/products/live-cell-imaging-analysis/live-cell-analysis-software
R (version 4.0.1)	R software	https://www.r-project.org/
Other		
AcroPrep 96 well 3.0 μm glass fiber/0.2 μm Bio-Inert membrane filter plate	Pall Laboratory	Cat# 5053
Odyssey CLx scanner	LI-COR	Cat# 9140
VCX 130 Vibra Cell sonicator	Sonics	Cat# CV18
MACSQuant VYB cytometer	Miltenyi	Cat# 130–096-116
4150 Tapestation System	Aglient Technologies	Cat# G2992AA
NextSeq2000	Illumina	Cat# 20038897
IncuCyte S3	Essen Biosciences	Cat# 4637
EVOS FL Auto 2 automated microscope	Thermo Fisher Scientific	Cat# AMAFD2000
Spark microplate reader	Tecan	Cat# 30124664
HiSeq 4000	Illumina	Cat# SY-401–1001

## Highlights

- The lethality of RNA Pol II inhibition results from active signaling, not passive mRNA decay
- Death is initiated by loss of hypophosphorylated (not actively elongating) RNA Pol IIA
- Genetic profiling reveals how loss of RNA Pol IIA is sensed and signaled to mitochondria
- Drugs with diverse annotated mechanisms owe their lethality to loss of RNA Pol IIA
