# Supplementary material for: RNA Pol II inhibition activates cell death independently from the loss of transcription
Source: Cell. Author manuscript; Available in PMC 2025 Sep 4. (PMC12406974; doi:10.1016/j.cell.2025.07.034)
Supplement: 8 [file NIHMS2100174-supplement-8.pdf]

CellPress
OPEN ACCESS

Supplemental figures

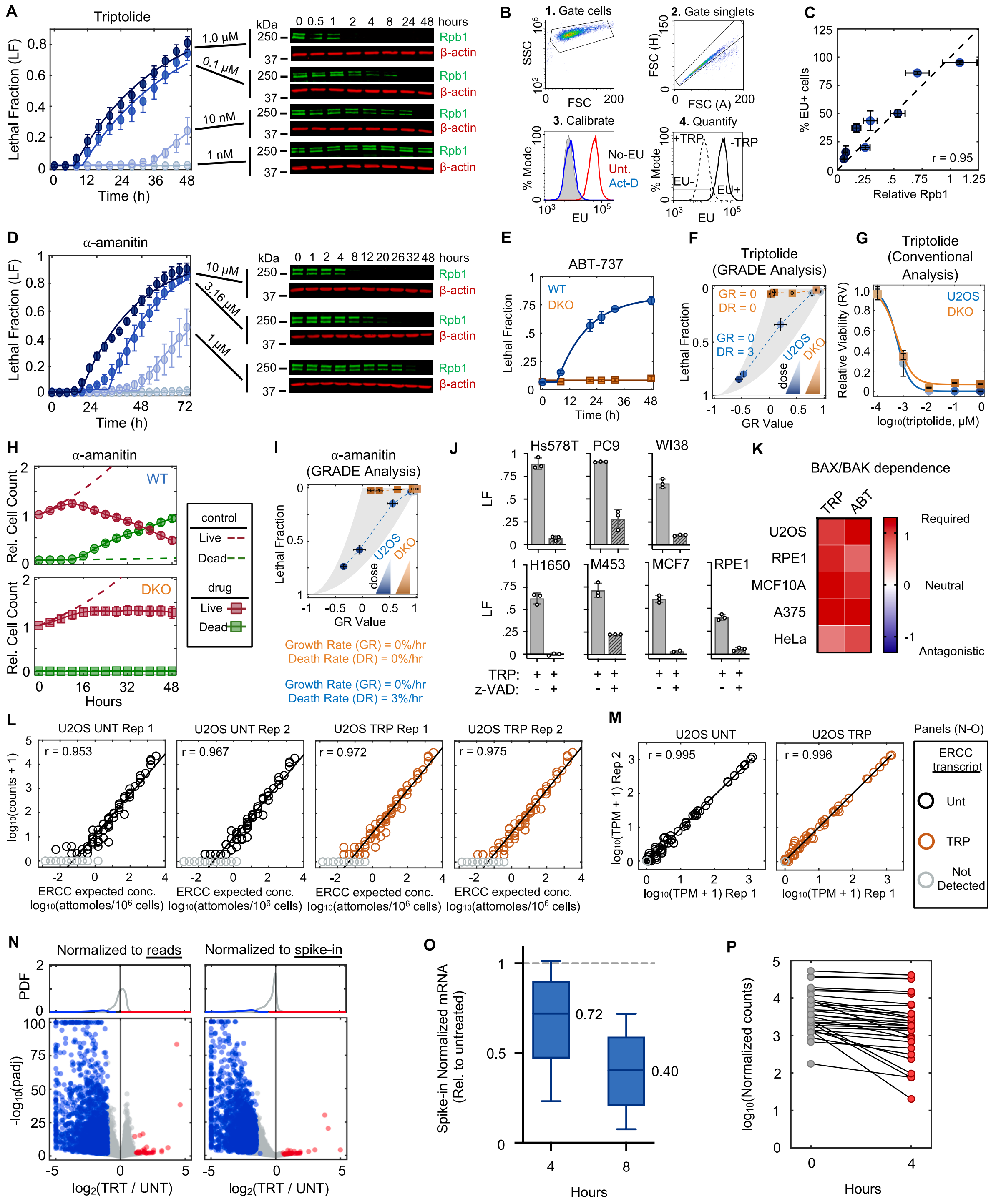

(legend on next page)

Figure S1. Transcriptional inhibition and associated death following exposure to RNA Pol II degraders, TRP, and α-amanitin, related to Figure 1

(A) (Left) LF in U2OS cells following varied doses of TRP, measured in STACK. (Right) Immunoblots of Rpb1 protein levels in U2OS cells following exposure to varied doses of TRP. Blots are representative of three independent biological replicates.

(B) Approach for measuring incorporation of 5-ethynyl uridine (EU) into newly synthesized RNAs using flow cytometry. Following labeling of nascent RNA via incubation with EU, cells are fixed, and labeled RNA is ligated to a fluorophore using click chemistry. A high dose (1 μM) and 18-h incubation with the pan-RNA polymerase inhibitor Act-D is used as a control to define the signal associated with no active transcription (EU−).

(C) Correlation between Rpb1 protein loss (quantified by immunoblot) and nascent RNA loss (quantified by EU incorporation) following exposure to 1 and 0.1 μM TRP (at 1, 2, 4, and 8 h post drug addition). Dashed line, x = y. Pearson correlation coefficient is shown.

(D) As in (A), but for α-amanitin.

(E) LF kinetics in U2OS (WT) and U2OS$^{BAX-/-BAK1-/-}$ (DKO) cells following exposure to the apoptotic agent ABT-737 (31.6 μM), measured in FLICK.

(F) GRADE-based analysis of TRP at 48 h. Inference of growth (GR) and death (DR) rates (% per hour) for the highest dose (1 μM) are shown for both genotypes.

(G) TRP sensitivity in U2OS and DKO cells quantified using the conventional measure of drug sensitivity, relative viability (RV). RV was measured using the STACK assay 120 h after drugging.

(H) Live and dead cell kinetics following exposure to 10 μM α-amanitin.

(I) As in (F), but for α-amanitin.

(J) LF for a panel of cell lines following 72-h treatment with 1 μM TRP, with or without co-treatment with 50 μM z-VAD, measured using FLICK.

(K) Heatmap depicting BAX/BAK dependence for 1 μM TRP or 100 μM ABT-199, measured using FLICK.

(L–N) Normalization of RNA-seq data to polyadenylated ERCC spike-ins. (L) The expected molar amount of RNA for each of the 92 ERCC spike-in transcript standards compared with the empirically observed read counts for replicates of U2OS RNA-seq samples untreated (UNT) or treated with 1 μM TRP for 4 h. Gray circles denote ERCC transcripts with zero counts. The linear regression line is shown, along with the Pearson correlation coefficient. (M) Transcript abundance correlation between replicates for the 92 ERCC spike-in transcript standards (TPM, transcripts per million). (N) Spike-in normalization is required to measure mRNA on an absolute scale. RNA-seq profiling of mRNA expression changes following exposure to 1 μM TRP for 4 h. (Left) mRNA expression normalized to read depth. (Right) mRNA expression normalized to ERCC spike-ins. Genes are colored based on read depth normalization: gray, no change; blue, downregulated; red, upregulated.

(O) mRNA transcript abundance for the following 4 or 8 h of 1 μM TRP, measured using spike-in normalized RNA-seq. Boxplots depict the 10–90 percentile, and the median value is stated.

(P) mRNA expression following exposure to 1 μM TRP for apoptotic regulatory genes, measured using spike-in normalized RNA-seq. Unless otherwise stated, for all panels with error bars, data are mean ± SD, *n* = 3 independent biological replicates.

CellPress
OPEN ACCESS

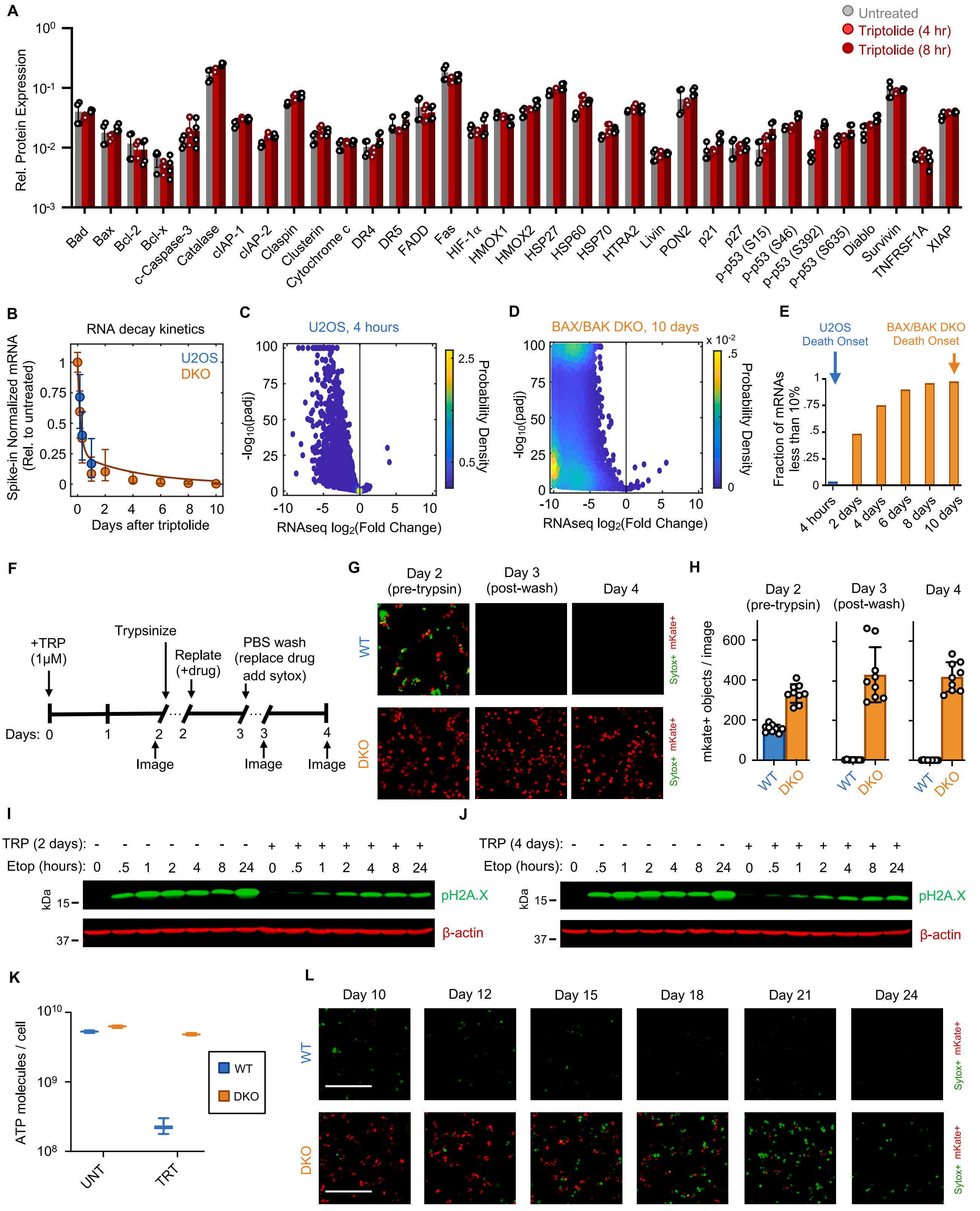

(legend on next page)

Figure S2. Viability of apoptotic-deficient cells following loss of RNA Pol II and loss of RNA, related to Figure 1

(A) Apoptotic protein expression measured using the proteome profiler apoptotic array for untreated U2OS cells and U2OS cells treated with 1 μM TRP for 4 or 8 h. Data are means ± SD of two independent biological replicates and two technical replicates, normalized to the highest expression protein (total pro-caspase-3). Significant differences between treated and untreated conditions for each epitope were determined using unpaired t tests with Welch correction, and the two-stage step-up method of Benjamini, Krieger, and Yekutieli was used to correct for multiple comparisons. No proteins were significantly decreased following TRP exposure, and phospho-p53 (S392) at 4 and 8 h and HSP60 at 8 h were modestly increased ($q < 0.05$).

(B) Decay kinetics for mRNAs in U2OS and BAX/BAK DKO (DKO) after 1 μM TRP exposure for the indicated times, measured using spike-in normalized RNA-seq. Datapoints indicate median, 25th, and 75th percentiles.

(C) Spike-in normalized mRNA fold changes in U2OS cells following 4 h of 1 μM TRP.

(D) Spike-in normalized mRNA fold changes in BAX/BAK DKO cells following 10 days of 1 μM TRP.

(E) Fraction of mRNAs that have decayed beyond 90% of untreated steady-state abundance. U2OS (blue) and BAX/BAK DKO cells (orange) are shown, and their respective death onset times are noted.

(F–L) Viability of BAX/BAK DKO cells during long-term exposure to TRP. (F) Experimental overview for testing the ability of apoptotic-deficient cells to reattach to plastic following trypsinization. (G) Representative images of TRP-treated cells before and after trypsinization and subsequent replating. (H) Quantification of mKate+ nuclei per image before and after trypsinization and subsequent replating, associated with (G). (I) Immunoblot depicting intact DDR signaling in both untreated and TRP-treated BAX/BAK DKO cells. Cells were first treated with or without 1 μM TRP for 2 days to induce RNA Pol II degradation, followed by the addition of 31.6 μM etoposide to induce DNA damage. pH2A.X (S139) levels were monitored over time following etoposide. (J) As in (I), except BAX/BAK DKO cells were exposed to TRP for 4 days. (K) ATP molecules per cell following a 4-day treatment with 1 μM TRP in U2OS or BAX/BAK DKO cells. Mean, minimum, and maximum values of $n = 60$ independent biological replicates are shown. (L) Long-term survival of BAX/BAK DKO cells following 1 μM TRP (associated with Figure 1F). Representative images for three independent biological replicates are shown.

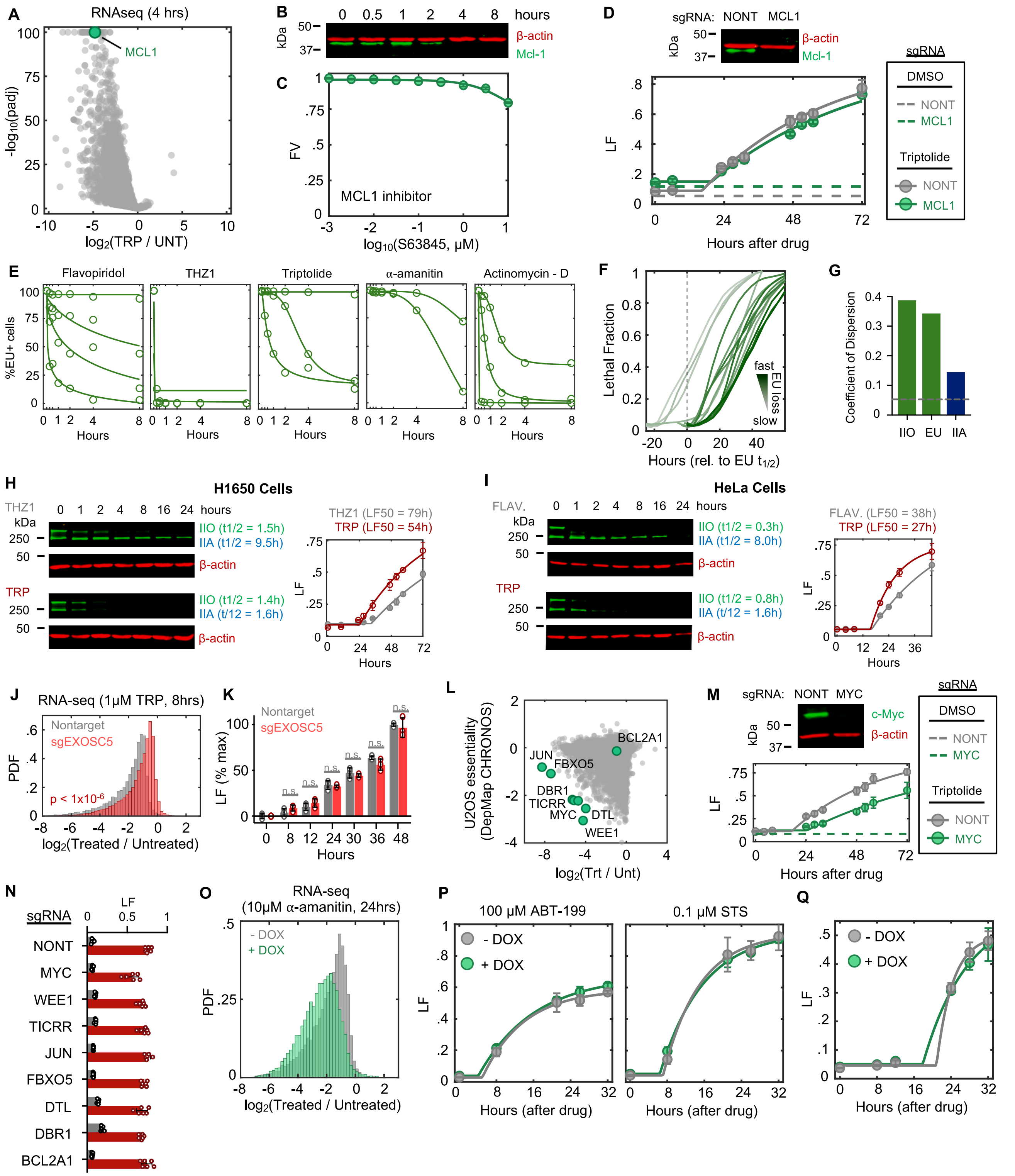

Figure S3. The lethality of transcriptional inhibition cannot be explained by loss of short-lived RNAs, related to Figures 2 and 3

(A–D) Rapid loss of MCL1 cannot explain lethality of TRP. (A) mRNA expression following 4-h exposure to 1 μM TRP in U2OS cells, highlighting the rapid loss of *MCL1*. (B) Immunoblot of Mcl-1 protein levels in U2OS cells following exposure to 1 μM TRP. (C) U2OS cells are insensitive to the Mcl-1 inhibitor S63845. FV measured after 72 h of drug exposure, measured in FLICK. Data are mean ± SD for $n = 3$ independent biological replicates. (D) Knockout of *MCL1* is not lethal in U2OS cells and does not impact the lethality induced by TRP. (Top) Immunoblot of Mcl-1 protein levels in U2OS cells expressing sgRNA targeting *MCL1* or

(legend continued on next page)

nontargeting sgRNA. (Bottom) LF kinetics between the two genotypes following exposure to TRP or vehicle (DMSO, 0.1%). LF kinetics were measured in FLICK. Protein lysates used for the immunoblot were taken from the same pool of cells used for LF measurements and were collected at the time of drugging (“0 h”).

(E–G) Quantifying variation in kinetics of RNA Pol II inhibition across transcriptional inhibitors using RNA Pol IIO and EU incorporation. (E) Measurement of nascent transcription across conditions in Figure 2, measured by flow cytometry. Ethynylcytidine is excluded, as it is itself CLICK-reactive. (F) Cell death kinetics for inhibitor conditions shown in (F), aligned to $t_{1/2}$ for loss of EU (dashed vertical line). Cell death was quantified using the STACK assay, and the mean of 3 independent biological replicates measured every 4 h is shown. (G) Coefficient of dispersion (CoD) of LF_{50} times for cell death kinetics aligned to loss of RNA Pol IIA (IIA), RNA Pol IIO (IIO), or RNA Pol II transcriptional activity (EU), as shown in Figures 2C, 2E, and S3F. Gray dashed line denotes the CoD for the “perfect” alignment, defined by arbitrarily shifting each kinetic curve such that maximum alignment is achieved, implemented using the MATLAB function alignsignals with method “risetime.”

(H and I) Example data from multiple cell lines that time to death and timing of transcriptional inhibition are not well-matched, but lethality data are well-matched to the timing of RNA Pol IIA loss. (H) RNA Pol IIO/A immunoblots and LF kinetics following 1 μM THZ1 or 1 μM TRP in H1650 cells. THZ1 and TRP cause RNA Pol IIO loss with similar kinetics, but THZ1 is much slower to degrade RNA Pol IIA. LF kinetics vary with RNA Pol IIA. (I) As in (H), but for 10 μM flavopiridol (FLAV.) or 1 μM TRP in HeLa cells. FLAV. and TRP cause RNA Pol IIO loss with similar kinetics, but FLAV. is much slower to degrade RNA Pol IIA. LF kinetics continue to vary with RNA Pol IIA.

(J and K) Effects of TRP on EXOSC5 knockout cells. (J) Histogram of spike-in normalized absolute mRNA fold changes following 8-h exposure to 1 μM TRP in U2OS cells expressing sgRNA targeting EXOSC5 or nontargeting sgRNA. Two-sided KS test *p* value is shown. (K) Comparison of LF levels between cell types in (J) at various time points following exposure to 1 μM TRP, measured using the FLICK assay. Data normalized to max LF in cells expressing nontargeting sgRNA. Wilcoxon rank sum *p* value shown (n.s. $p > 0.05$).

(L) Identification of short-lived mRNAs that continue to be rapidly lost in NUP93-KO cells and are also essential in U2OS. Essentiality in this context means essential for normal proliferation.

(M) LF kinetics in MYC-KO cells, compared with parent U2OS cells.

(N) LF in U2OS cells transiently transfected with sgRNA targeting genes highlighted in (L) and exposed for 72 h to DMSO (gray) or 1 μM TRP (red). Mean ± SD of 6 biological replicates shown.

(O–Q) Characterization of the RNA Pol II switchover system. (O) Histogram of spike-in normalized absolute mRNA fold changes following 24-h exposure to 10 μM α-amanitin. (P) LF kinetics following exposure to general apoptotic agents in RNA Pol II switchover cells with or without DOX-induced expression of RPB1-N792D-ΔCTD. STS, staurosporine. (Q) LF kinetics in cells with or without expression of an α-amanitin-sensitive *RPB1*-ΔCTD transgene, treated with 10 μM α-amanitin. For all panels with error bars, data are mean ± SD shown, $n = 3$ independent biological replicates unless otherwise noted.

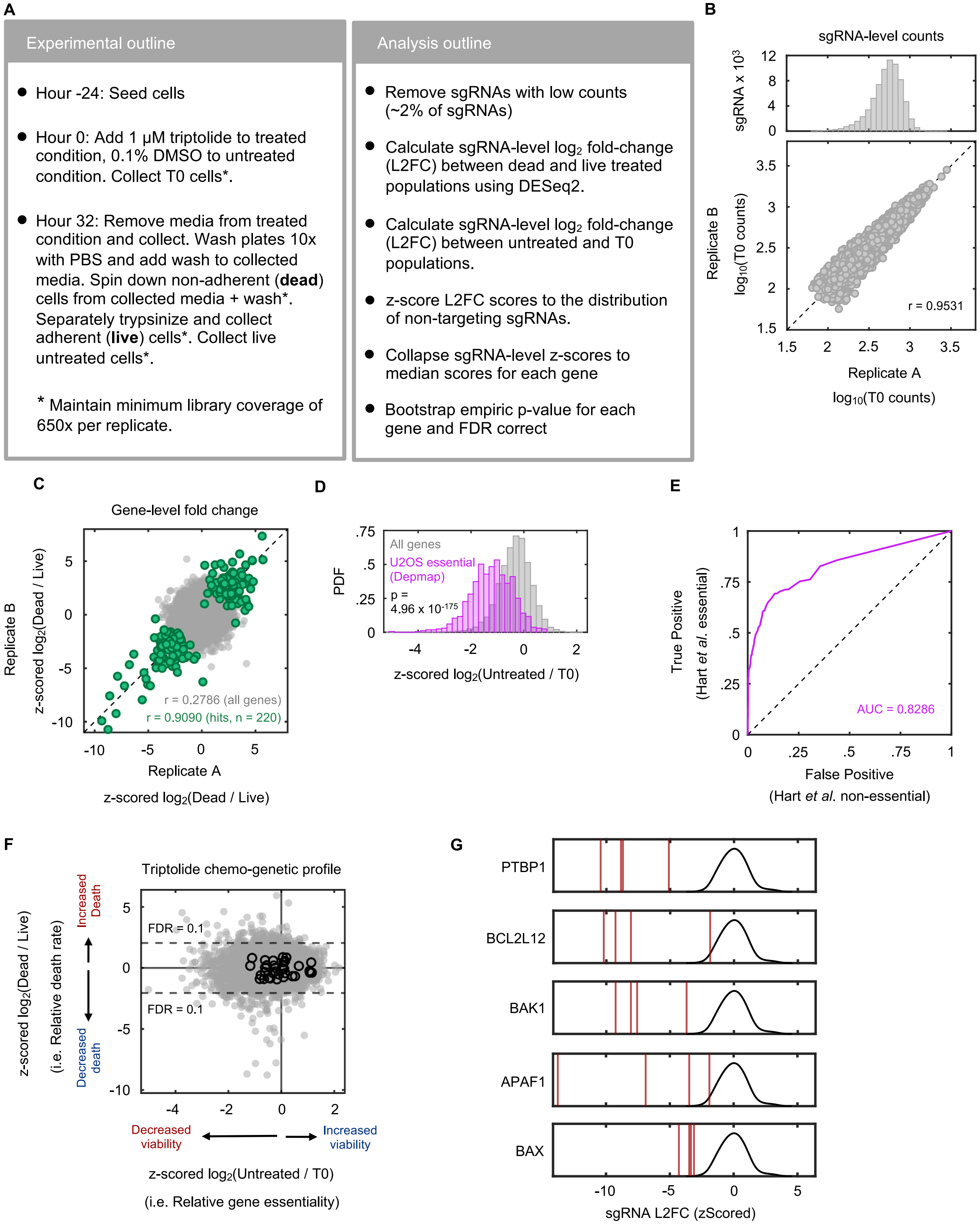

(legend on next page)

Figure S4. Chemogenetic profiling strategy and quality assessment, related to Figure 4

(A) Overview of experimental and analytical process for the chemogenetic screen.

(B) (Top) Representative example of sgRNA count distribution for replicate A of the T0 condition. (Bottom) Representative example of correlation of sgRNA counts between two replicates of the same condition. Dashed line, x = y. Pearson correlation coefficient is shown.

(C) Correlation of gene-level L2FC scores between two replicates treated with TRP.

(D) Distribution of gene-level L2FC scores for essential genes versus all genes in untreated versus T0 comparison. Two-sided KS test *p* value is shown.

(E) ROC curve depicting the sensitivity and specificity of the untreated versus T0 screening comparison to classify previously established essential and non-essential genes.

(F) Conceptual overview of metrics comprising the chemogenetic profile of TRP. The *x* axis describes the effect a gene knockout has on the viability of a cell in untreated conditions. The *y* axis describes the effect a gene knockout has on the cell death rate in the context of TRP. Scores for all genes (gray, filled) and non-targets (black, empty) are shown.

(G) sgRNA-level log_2 fold changes (dead population/live population) for PTBP1 and BCL2L12 in the context of 1 μM TRP. BAK1, APAF1, and BAX are core apoptotic regulators and act as controls. Fold changes for 4 sgRNAs per gene (red vertical lines) were *Z* scored to the distribution of nontargeting guides (black).

CellPress
OPEN ACCESS

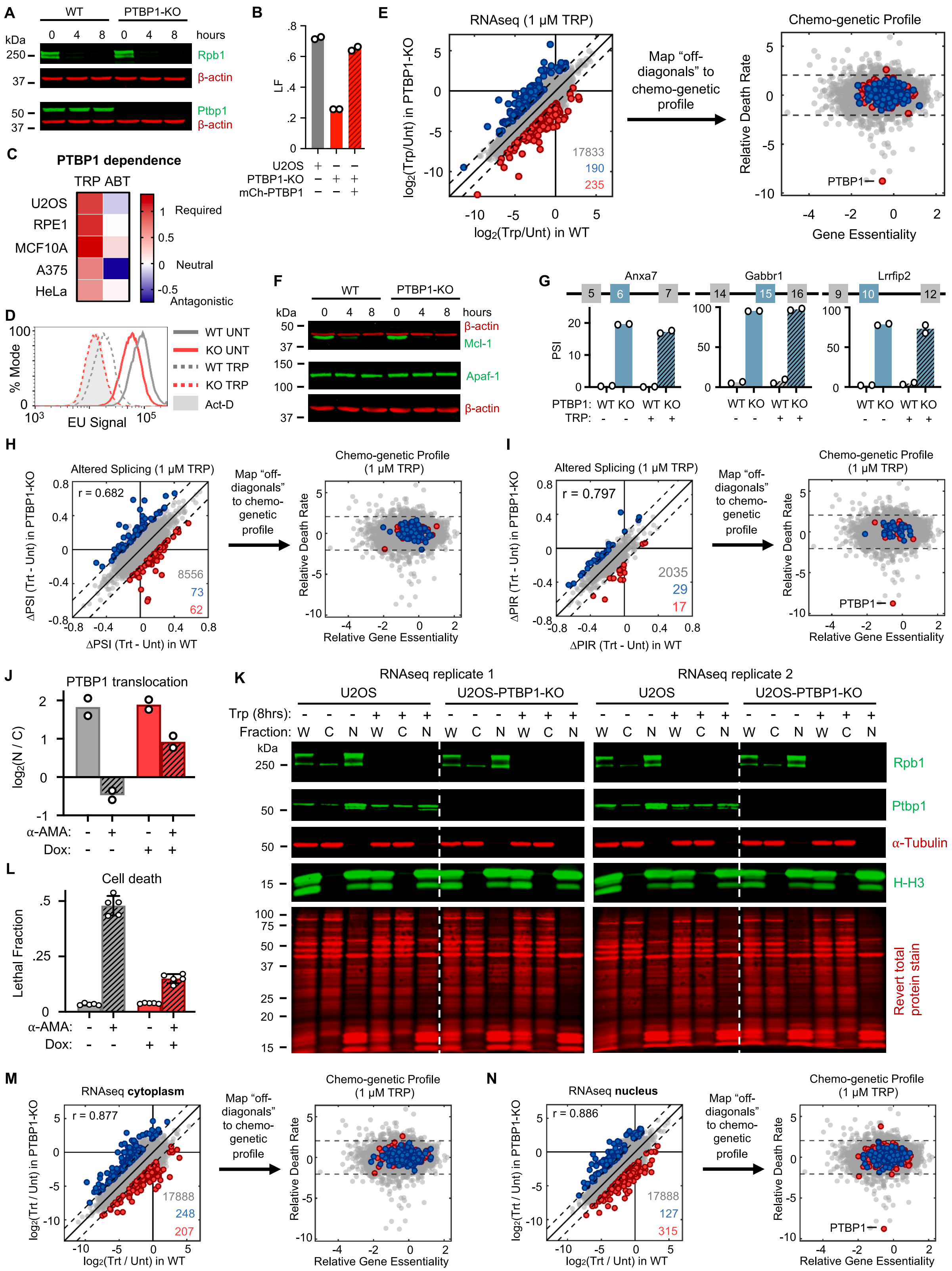

(legend on next page)

Figure S5. Validating PTBP1-dependent death following RNA Pol II degradation and evaluating the contribution of known functions of PTBP1, related to Figure 5

(A) Immunoblots for Rpb1 protein levels following exposure to 1 μM TRP in U2OS and U2OS-PTBP1-KO. PTBP1-KO has no effect on RNA Pol II degradation.
(B) LF following 1 μM TRP exposure for 48 h in U2OS cells or PTBP1-KO cells, with or without expression of mCherry-PTBP1. Exogenous expression of mCherry-tagged PTBP1 restores TRP sensitivity in PTBP1 knockout cells.
(C) Heatmap depicting PTBP1-dependence for 1 μM TRP or 100 μM ABT-199, measured using FLICK.
(D) EU incorporation into nascent RNA in U2OS and U2OS-PTBP1-KO. Cells treated with or without 1 μM TRP for 8 h prior to EU labeling. Populations are representative of 3 biological replicates. PTBP1-KO has no effect on the TRP-induced loss of nascent transcription.
(E) (Left) RNA-seq data identifying rare outlier genes whose expression is decreased more (red) or less (blue) in PTBP1-KO cells compared with WT cells following TRP. The threshold for an "outlier" is depicted by the dashed lines, denoting 2.5 standard deviations from the identity line (x = y). Number of outliers shown. (Right) Mapping of expression outliers onto the chemogenetic profile for TRP.
(F) Immunoblots for Mcl-1 and Apaf-1 protein levels following exposure to 1 μM TRP in U2OS and U2OS-PTBP1-KO cells.
(G–I) The role of PTBP1 in TRP-induced lethality is unrelated to alternative splicing. (G) A subset of previously annotated PTBP1-dependent exon exclusion events. PSI, percent spliced in for the blue-colored exon. Data are with or without 1 μM TRP for 8 h. (H) (Left) Comparison of drug-induced splicing changes in U2OS and U2OS-PTBP1-KO cells, measured 8 h following treatment with 1 μM TRP. Data include alternative 3′ splice site, alternative 5′ splice site, and alternative exon usage. Dashed lines denote 2.5 standard deviations from the identity line (x = y), and off-diagonals are highlighted. (Right) Mapping of outliers onto the chemogenetic profile for TRP. (I) As in (E), but specifically for intron retention (IR).
(J–N) Evaluation of PTBP1 nuclear export following RNA Pol II degradation. (J) Quantification of the nuclear-to-cytoplasmic ratio of PTBP1 protein before and after exposure to 10 μM α-amanitin for 12 h in RNA Pol II switchover cells, measured using immunoblotting. (K) PTBP1 translocation following TRP exposure. These samples were also used for RNA-seq of nuclear and cytoplasmic fractions. Protein samples were simultaneously obtained from the identical cell lysates used to isolate and sequence mRNA. W, whole-cell lysate; C, cytoplasmic fraction; N, nuclear fraction. (L) LF measured 32 h after exposure to 10 μM α-amanitin in RNA Pol II switchover cells. Mean ± SD shown, *n* = 5 independent biological replicates. (M) (Left) Comparison of drug-induced expression changes of cytoplasmic mRNA in U2OS and U2OS-PTBP1-KO cells, following 8-h exposure to 1 μM TRP. Dashed lines denote 2.5 standard deviations from the identity line (x = y), off-diagonals are highlighted, and the Pearson correlation coefficient of all genes is shown. (Right) Mapping of outliers onto the chemogenetic profile for TRP. (N) As in (M), but specifically for nuclear mRNAs.

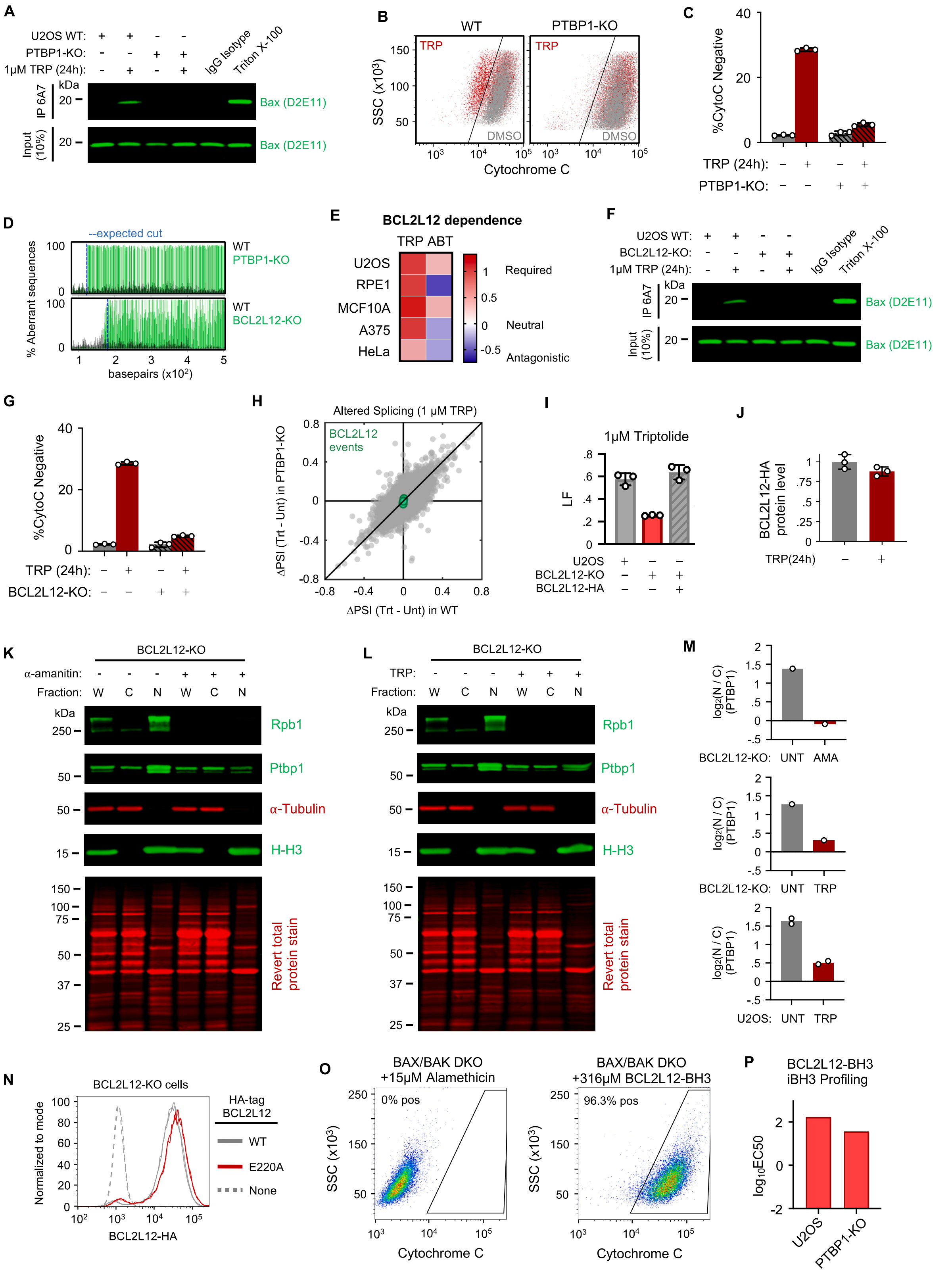

(legend on next page)

Figure S6. Validating BCL2L12-dependent death following RNA Pol II degradation, related to Figure 6

(A) BAX activation following TRP in U2OS WT and PTBP1-KO cells, measured by immunoprecipitating active BAX using the conformation-specific antibody 6A7. Concentration-matched isotype IP and 6A7-IP in Triton X-100 detergent denote negative and positive controls, respectively.
(B) Cytochrome *c* release in WT (left) and PTBP1-KO (right) cells exposed to TRP or DMSO for 24 h, measured in flow cytometry. Cells were co-treated with 50 μM z-VAD to reduce dead cell loss. Data are representative of 3 independent biological replicates.
(C) Quantification of cytochrome *c* release depicted in (B). Mean ± SD of 3 independent biological replicates shown.
(D) TIDE analysis of PTBP1 and BCL2L12 knockout U2OS clones, validating sgRNA activity. No WT sequence was identified in the clones.
(E) Heatmap depicting the dependence of cell death induced by 1 μM TRP or 100 μM ABT-199 on BCL2L12, measured using FLICK.
(F) BAX activation following TRP in U2OS WT and BCL2L12-KO cells, as in (A).
(G) Quantification of cytochrome *c* release in WT and BCL2L12-KO cells following exposure to DMSO or 1 μM TRP, measured using flow cytometry. Mean ± SD of 3 independent biological replicates are shown.
(H) Comparison of drug-induced splicing changes in U2OS (*x* axis) and U2OS-PTBP1-KO (*y* axis) cells, measured 8 h following treatment with 1 μM TRP. Data include alternative 3′ splice site, alternative 5′ splice site, and alternative exon usage. Splice events annotated to BCL2L12 are highlighted.
(I) C-terminal HA-tagged BCL2L12 was expressed in U2OS-BCL2L12-KO cells, and cell death following 42 h of 1 μM TRP was measured using FLICK. Mean ± SD of 3 independent biological replicates shown.
(J) BCL2L12-HA protein levels before and after 24 h of TRP, measured by quantitative immunoblotting using anti-HA. Mean ± SD of 3 independent biological replicates shown.
(K) PTBP1 translocation following 12 h of 10 μM α-amanitin in WT and BCL2L12-KO U2OS cells. W, whole-cell lysate; C, cytoplasmic fraction; N, nuclear fraction.
(L) As in (K), but 8 h of 1 μM TRP.
(M) Quantification of fractionation data in (K) and (L). PTBP1 fractionation in U2OS cells treated with TRP is shown for comparison.
(N) Comparison of BCL2L12 protein expression in BCL2L12-KO cells exogenously expressing WT or mutated (E220A) BCL2L12, measured in flow cytometry.
(O) Cytochrome *c* release in U2OS-BAX-BAK-DKO cells following a 1-h exposure with the depolarizing agent alamethicin or 316 μM BCL2L12-BH3-like peptide, measured using the iBH3 assay.
(P) The EC_{50} dose for cytochrome *c* release following exposure to the BCL2L12-BH3-like peptide, in WT and PTBP1-KO U2OS cells, measured using the iBH3 assay. EC_{50} values were fit to a 4-term Hill equation fit to 3 independent biological replicates.

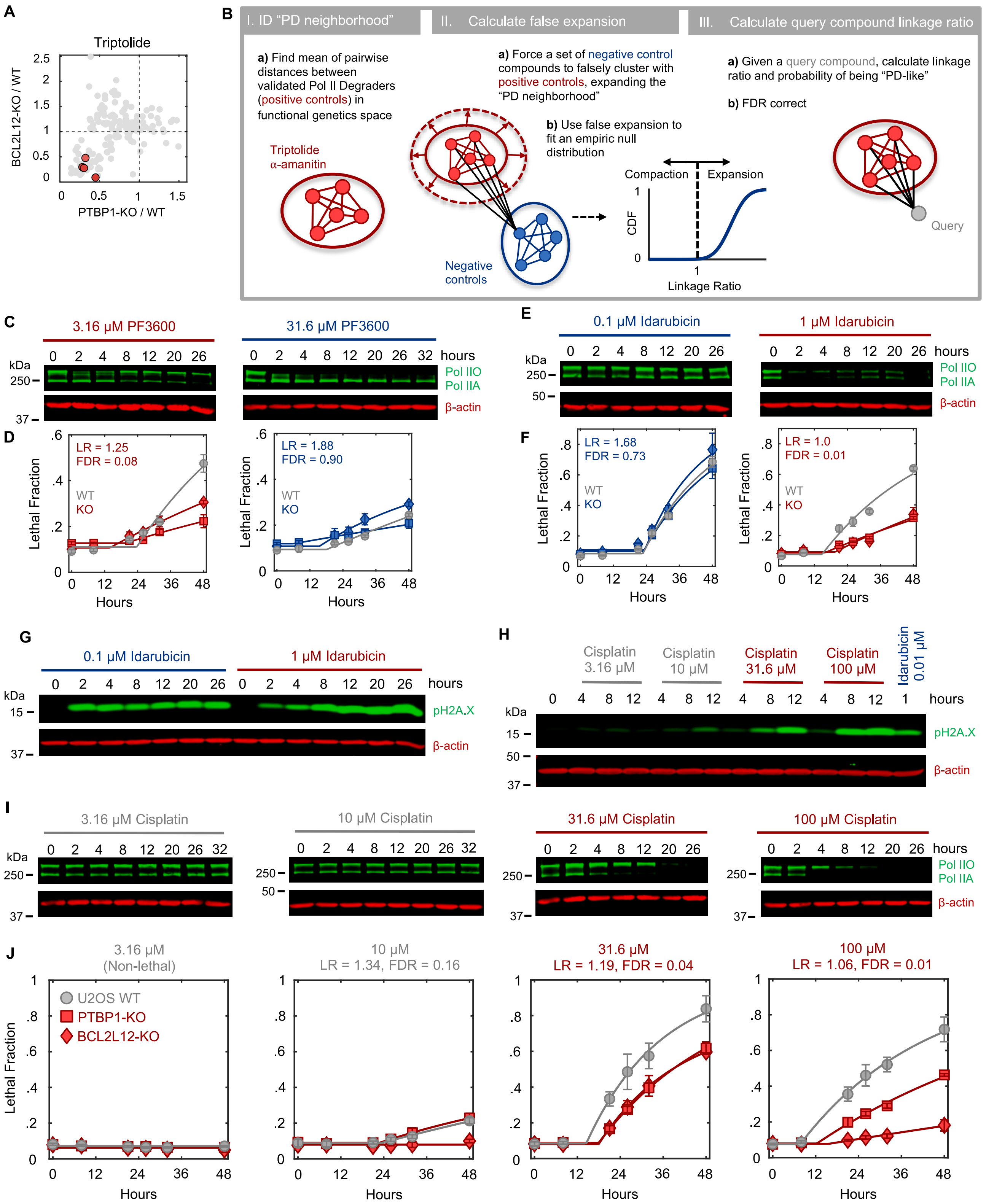

Figure S7. A statistical classifier to identify PD-like compounds, related to Figure 7

(A) Map of the functional effect of PTBP1 and BCL2L12 knockout on the cell killing of each drug-dose pair (gray points). TRP highlighted.

(B) Conceptual overview of the probabilistic nearest neighbors-based classification approach (adapted from Pritchard et al.[59]).

(legend continued on next page)

(C and D) Response to PF3600 at a PD-like (red) and non-PD-like (blue) dose. (C) Rbp1 levels following 3.16 or 31.6 μM PF3600. (D) LF kinetics for 3.16 or 31.6 μM PF3600. Data are from U2OS cells (gray circles), PTBP1-KO cells (squares), and BCL2L12-KO cells (diamonds), measured using FLICK. Linkage ratios and associated FDR values are shown.

(E and F) As in (C) and (D) for 0.1 and 1 μM idarubicin.

(G) Similar DDR signaling, as measured by pH2A.X levels in immunoblot, is seen at both doses of idarubicin shown in (E) and (F).

(H) Immunoblot of p-H2A.X levels across doses of cisplatin. All doses of cisplatin that induce appreciable amounts of p-H2A.X signaling are PD-like. A low, and non-lethal, dose of the DNA-damaging agent idarubicin is shown for comparison.

(I and J) As in (C) and (D), but for varied doses of cisplatin. For all panels with error bars, data are mean ± SD, *n* = 3 independent biological replicates.
